# Microalga Broths Synthesize Antibacterial and Non-Cytotoxic Silver Nanoparticles Showing Synergy with Antibiotics and Bacterial ROS Induction and Can Be Reused for Successive AgNP Batches

**DOI:** 10.3390/ijms242216183

**Published:** 2023-11-10

**Authors:** Carlos Pernas-Pleite, Amparo M. Conejo-Martínez, Paloma Fernández Freire, María José Hazen, Irma Marín, José P. Abad

**Affiliations:** 1 Department of Molecular Biology, Faculty of Sciences, Biology Building, Autonomous University of Madrid, Cantoblanco, 28049 Madrid, Spain; 2 Department of Biology, Faculty of Sciences, Biology Building, Autonomous University of Madrid, Cantoblanco, 29049 Madrid, Spain

**Keywords:** green synthesis, silver nanoparticles, antibacterial activity, cytotoxicity, ROS production, antibacterial synergistic activity, microalgae, acid-tolerant, Tinto River, AgNPs from recycled broths

## Abstract

The era of increasing bacterial antibiotic resistance requires new approaches to fight infections. With this purpose, silver-based nanomaterials are a reality in some fields and promise new developments. We report the green synthesis of silver nanoparticles (AgNPs) using culture broths from a microalga. Broths from two media, with different compositions and pHs and sampled at two growth phases, produced eight AgNP types. Nanoparticles harvested after several synthesis periods showed differences in antibacterial activity and stability. Moreover, an evaluation of the broths for several consecutive syntheses did not find relevant kinetics or activity differences until the third round. Physicochemical characteristics of the AgNPs (core and hydrodynamic sizes, Z-potential, crystallinity, and corona composition) were determined, observing differences depending on the broths used. AgNPs showed good antibacterial activity at concentrations producing no or low cytotoxicity on cultured eukaryotic cells. All the AgNPs had high levels of synergy against *Escherichia coli* and *Staphylococcus aureus* with the classic antibiotics streptomycin and kanamycin, but with ampicillin only against *S. aureus* and tetracycline against *E. coli*. Differences in the synergy levels were also dependent on the types of AgNPs. We also found that, for some AgNPs, the killing of bacteria started before the massive accumulation of ROS.

## 1. Introduction

Antimicrobial resistance to antibiotics is one of the more acute human threats that has emerged in the last century [1]. Drug-resistant infections are increasing, and researchers and international agencies estimate 10 million deaths per year by 2050, with high economic losses [2]. Therefore, searching for new antimicrobials is essential for global healthcare. Among these, metal-based drugs are promising to help overcome antibiotic resistance, specifically nanoparticles, with those based on silver being the most studied [3,4,5,6,7].

Besides all the properties of silver ions, also in the form of Ag^0^ nanoparticles, silver shows tremendous potential as an antibacterial agent [8,9,10,11], currently used in numerous consumer and medical products [12,13,14]. However, silver nanoparticles (AgNPs) have several drawbacks that must be studied [15]. Specifically, cytotoxicity is one of the main problems when using AgNPs. The toxicity of AgNPs has been investigated in human or other mammal cells and other organisms [16,17,18,19,20]. Several studies have evaluated the toxicity of AgNPs obtained by different methods [6], observing from low [21,22] to high [23] toxicity levels.

Many methods for AgNP synthesis have been developed based on chemical, physical, or biological protocols. The former two are advantageous because the size and the shape of the produced AgNPs are usually more regular, although their shortfalls are their toxicity, environmental concerns, and higher costs [24,25]. Biological methods are more advisable and greener because they are usually cheaper, use nontoxic agents, and do not require sophisticated equipment [5,26]. Biological procedures include cell-free methods, using extracts or culture broths, or cell-dependent methods, both intracellular (AgNPs formed inside the cells) and extracellular (AgNPs formed outside the cells) syntheses. The methods not requiring extraction procedures are cheaper and operationally simpler to perform. Extraction operations are needed when using macroorganisms such as plants, macroalgae, or animals, or microorganisms such as bacteria, fungi, or microalgae, in cell-dependent synthesis, but not when using culture broths. Collecting wild organisms from nature is not recommended because of environmental concerns; it is more appropriate to culture the living organisms when the massive production of AgNPs is required. From this, the use of culture broths is advantageous. The activity of the AgNPs will depend on the biological material and method used [24,27,28].

Microalgae are eukaryotic phototrophic microorganisms [29], widely studied as green bio-factories, producing different organics and used in biotechnological applications to produce compounds or for use as biomass [30]. One application for extracts or culture broths of microalgae could be the production of AgNPs. Many reports exist of AgNP synthesis by photosynthetic organisms [31], including plants (the most frequent) [28,32], algae (both macroalgae and microalgae) [33,34,35,36,37,38,39], and cyanobacteria [38,40]. AgNP production using microalgae is quite advantageous and sustainable [37], since, from their photoautotrophic growth, the media used to culture these organisms do not require the addition of organic chemicals. Moreover, extraction steps would not be needed if the broths from the microalgae culture were used for AgNP synthesis, thus simplifying the protocols and cheapening the production costs. However, using microalgae culture broths is less common than using cell extracts [31,34,35,36,38,39]. In addition, the culture of microalgae for biomass obtention to be employed for uses such as animal feeding and dyes or biofuel production [33], among others, is costly due to the processing charges, but it is considered an element of the circular economy and their use may help in the climate change context [41] and in sustainable development [42]. Thus, using broths from these cultures as subproducts of the principal process for AgNP production, as another additional application, might help to decrease overall costs and potentiate the use of microalgae. Microalgae-mediated nanomaterials present enormous potential for large-scale production, with promising applications in different sectors [34]. Examples of microalgae species already reported to be useful for AgNP production include *Chlamydomonas reinhardtii* [43], *Desmodesmus abundans* [44], *Parachlorella kessleri* [45], various chlorophytes and charophytes [35], and different isolates of *Botryococcus*, *Chlorella*, *Coelastrum*, and *Scenedesmus*, among others [38]. Since the physicochemical characteristics and biological properties of nanoparticles depend on the organism and production methods used, the search for new microalgae able to produce AgNPs with different applications—for instance, antimicrobial activity—is highly recommended.

Besides employing AgNPs alone as antimicrobials, their combined use with classical antibiotics may be another means of overcoming the antimicrobial resistance of pathogenic bacteria [46,47,48]. Synergistic behavior would allow the use of lower concentrations of both agents, the antibiotics and the AgNPs, with an overall better effect. Furthermore, using AgNPs in combination with lower concentrations of antibiotics could permit the employment of some classical antibiotics currently in disuse owing to bacterial resistance or toxic effects [49,50,51,52,53,54].

The mechanism for AgNP antimicrobial activity seems not unique, and different possible effects of these materials have been proposed [55,56,57]. Among these, the generation of reactive oxygen species (ROS) in bacteria is an effect frequently reported.

Aside from their use as antimicrobials, many other applications of AgNPs have been claimed, such as those in the biomedical field (as anticancer or antiviral drugs, or as sensors, among others) [58], but also in environmental applications (wastewater treatment, metal remediation, dye degradation) [59] and in physics (electronics, optics) [58,60], and new developments will probably emerge in the future.

In this study, we wished to use culture broths from an acid-tolerant microalga, not previously used, to test whether they could be employed for AgNP production, since most previous studies, using other biological materials and other microalgae, have preferentially used extracts. For broth obtention, we wished to vary the culture conditions, such as the pH (4 and 7) and culture media composition (media with chloride salts or without them), sampling from the early and late growth phases, to test whether these different conditions could produce AgNPs with various structures and properties. We determined their physicochemical characteristics, antibacterial activity, and cytotoxicity on human cells to assess their safety in application. Since one of the most rewarding applications of AgNPs could be in combination with classic antibiotics, we also sought to evaluate their capability to act synergistically with seven common antibiotics. We also obtained insights about the AgNPs’ characteristics at different times during their synthesis and studied the possibility of reusing the broths for successive AgNP syntheses. Finally, ROS production, one of the most frequently observed mechanisms involved in the antibacterial effects of AgNPs, was studied concerning its possible correlation with the bacterial killing activity of the AgNPs.

## 2. Results

### 2.1. Microalga Cultures and Growth Curves

**Figure 1:**
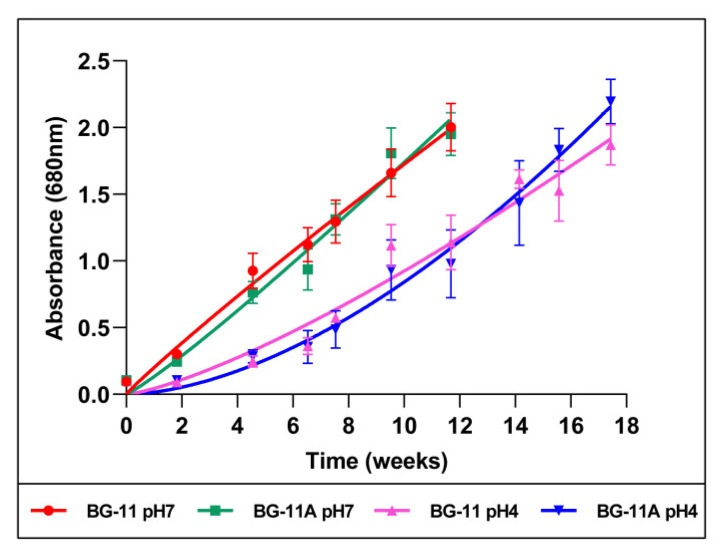
Microalga growth curves in two media and at two pHs.

A Chlorophyta microalga was previously isolated from the Tinto River estuarian waters. This river is an acidic habitat, and the microalga was acid-tolerant since it could grow at pH 4 and pH 7 (Figure 1). Four different culture conditions, corresponding to two pHs and two media, were used to culture the microalga and sampling broths at two growth phases, thus generating eight broths for their use in AgNP synthesis. Growth curves showed differences depending mainly on the culture medium pH, with the growth at an acidic pH being slower than that at a neutral pH. Since previous studies by others and our group have shown an effect of the presence of chloride on AgNP synthesis [61,62,63], a medium without Cl^−^ (BG-11A) was designed to prevent the formation of AgCl during the AgNP synthesis. Thus, the difference in composition of the two media used was that the chloride salts in the regular BG-11 medium were replaced by equimolecular concentrations of other salts of the same cations (see Section 4).

### 2.2. Biosynthesis of Silver Nanoparticles

**Figure 2:**
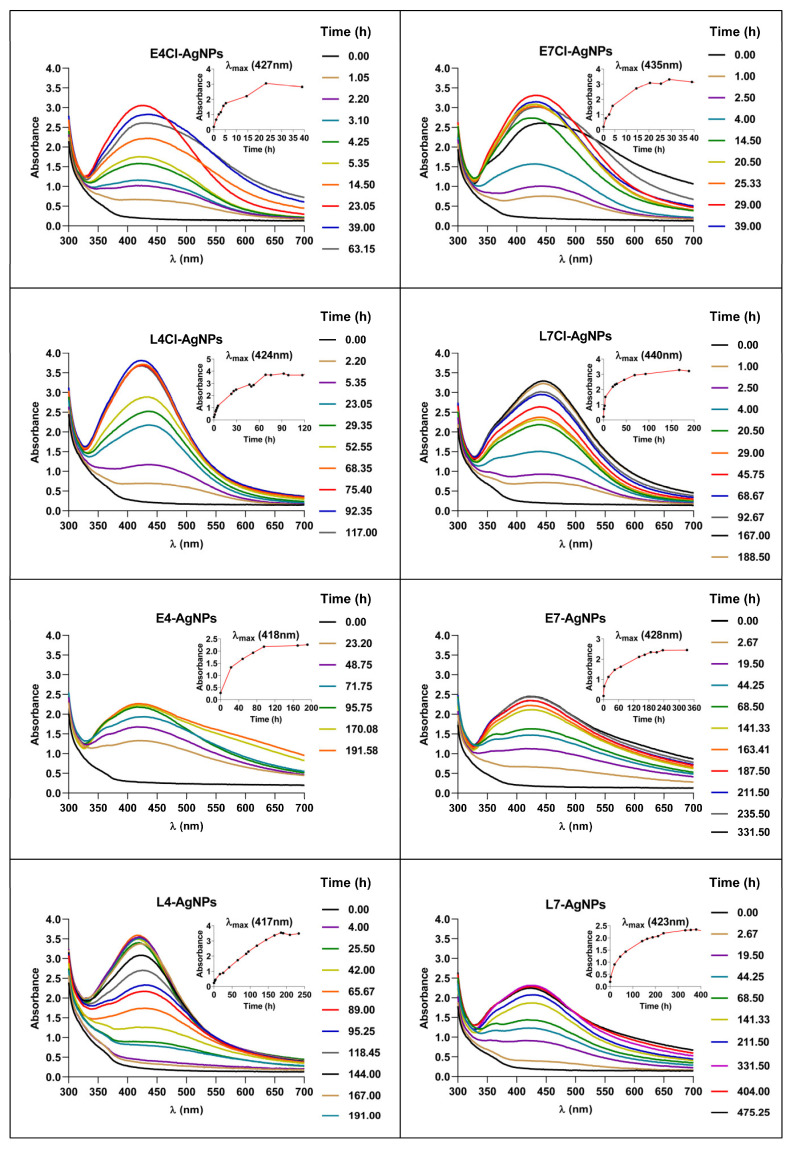
Kinetics of AgNP synthesis followed by UV–Vis spectroscopy. Graphs inserted in the upper right corner of each panel show the kinetics of synthesis from the increase in absorbance at the corresponding λ_max_.

The microalga culture broths, conveniently pre-treated, were mixed with ${AgNO}_{3}$ and incubated in the conditions indicated in the Section 4. The production of AgNPs, initially detected by the browning of the reaction mix, was spectroscopically followed during the reaction (Figure 2) (see naming of the different AgNPs in the Section 4). Spectra showed a time-dependent absorbance increase, with a peak at wavelengths between 417 nm and 440 nm, depending on the broth used for the synthesis. The kinetics of the reaction based on the absorbance at the maximum absorbance wavelength (λ_max_) indicated stabilization at incubation times between 24 and 400 h approximately, depending on the used broth. After reaching the kinetics plateau, further incubation led to changes in the spectra compatible with some aggregation of the nanoparticles. AgNPs were collected from the reaction mixes for their characterization before any sign of nanoparticle aggregation occurred.

The kinetics of AgNP synthesis reported here show relevant differences related to the media composition (with or without Cl^−^). The speed of AgNP production was higher for broths containing Cl^−^ (Figure 2). Concerning the pH of the culture media used for the microalga growth, the speed of synthesis was higher for pH 4 than for a neutral pH in similar conditions (Figure 2). The third variable in the preparation of different broths was the growth phase of the microalga cultures. Broths from the early growth phase produced faster reactions than those from the late one in similar conditions, reaching, for the early-phase nanoparticles, maximum production in the range of 24 h for the E4Cl-AgNPs to 160 h for the E7-AgNPs. The late-phase broths needed longer reaction times, in the range of 70–230 h, with the lowest value for L4Cl-AgNPs and the highest for L7-AgNPs. Broths from late-phase growth, in most cases, needed almost twice the time for maximum production (Figure 2).

#### 2.2.1. Synthesis Kinetics and Stability after Washing of AgNPs Produced at Different Times of the Synthesis Reaction

**Figure 3:**
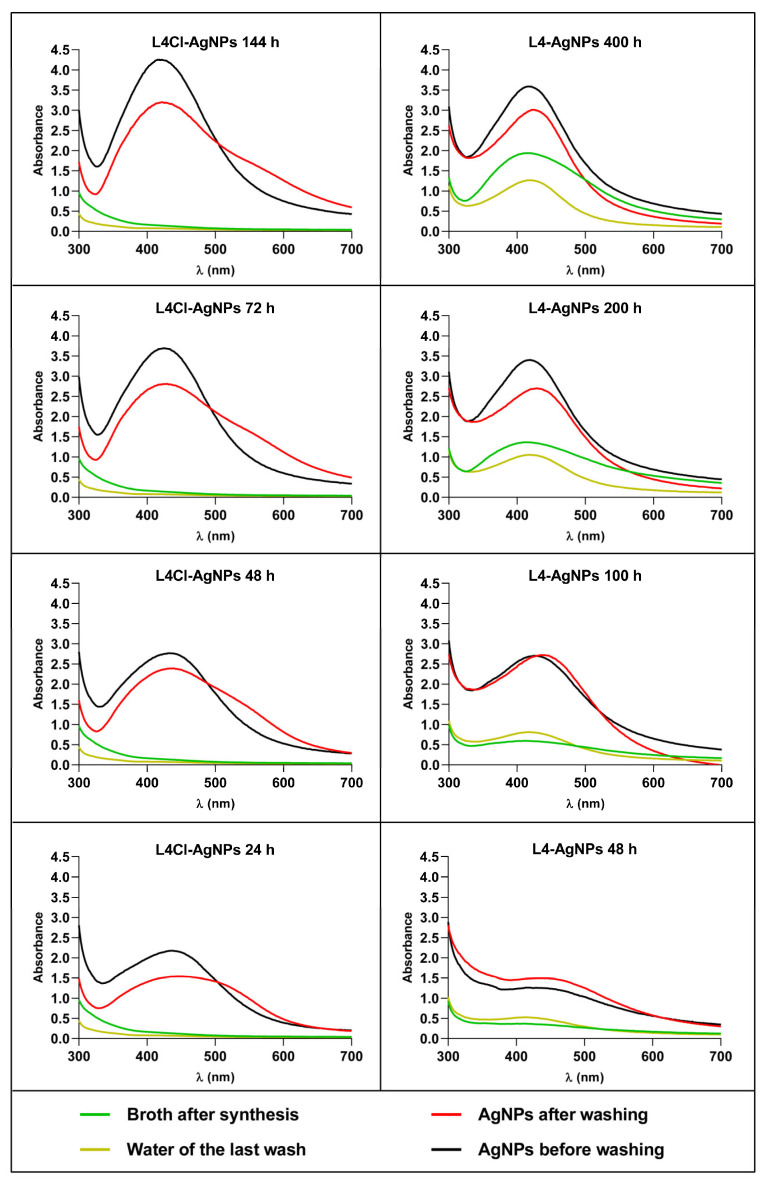
UV–Vis spectra of AgNPs harvested at different times of synthesis before and after washing.

To obtain insights into the process of AgNP synthesis, we analyzed the reaction mix over time for two of the AgNPs as models, L4Cl-AgNPs and L4-AgNPs, and purified the produced AgNPs at several time points in the reaction. The spectra’s shape showed no relevant differences but showed increasing absorbance over time, indicating a corresponding increase in the AgNPs’ concentration (Figure 2). The AgNPs collected at each time point and washed showed some spectral changes in the washed ones with respect to their original spectra (Figure 3), such as the widening of the UV–Vis spectra for the L4Cl-AgNPs, but not for the L4-AgNPs. These changes can be considered a sign of the lower stability of the L4Cl-AgNPs. When comparing the spectra of the AgNPs collected at different reaction times, the spectra showed a decreasing effect of washing at longer times in the case of the L4Cl-AgNPs. L4-AgNPs did not show this behavior. The residual waters from the washes showed almost flat spectra for L4Cl-AgNPs at the studied times. However, L4-AgNPs showed an absorbance increase at λ_max_ inversely correlating with their collection time from the reaction mix. This absorbance corresponds to the AgNPs that were not sedimented during the centrifugation step of the standard washing process, which remained in the washing water.

#### 2.2.2. Kinetics of Successive AgNP Syntheses from the Same Broth

**Figure 4:**
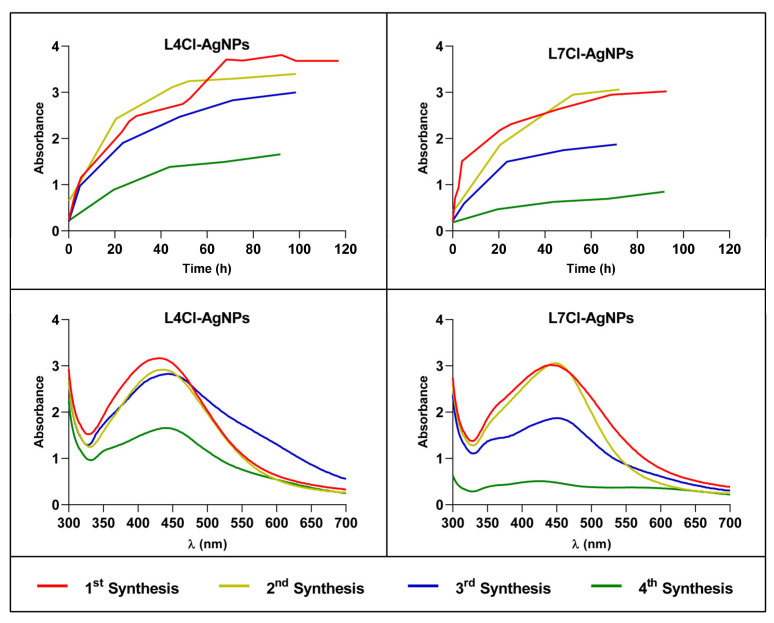
Analysis of AgNPs obtained by successive syntheses reusing broth. Upper panels: synthesis kinetics; lower panels: UV–Vis spectra of the AgNPs from the consecutive batches.

To test the possibility of reusing broths for several successive AgNP batches’ production, up to four consecutive synthesis reactions of nanoparticles were set using L4Cl-AgNPs and L7Cl-AgNPs as models. The kinetics did not significantly change in the second synthesis, but some differences appeared in the third and fourth rounds. These showed a decrease in the synthesis speed, reaching lower production. This effect was higher in the case of the L7Cl-AgNPs (Figure 4, upper panels), producing nanoparticles in lower yields. The AgNPs’ spectra (Figure 4, lower panels) suggested differences in structure and stability that rose in the two last rounds of synthesis. AgNPs from the third synthesis seemed to be less stable than those of the second one but more stable than those from the fourth one.

### 2.3. Characterization of Biosynthesized AgNPs

#### 2.3.1. Physicochemical Properties

##### Elemental Composition of AgNPs by Total Reflection X-ray Fluorescence (TXRF)

**Figure 5:**
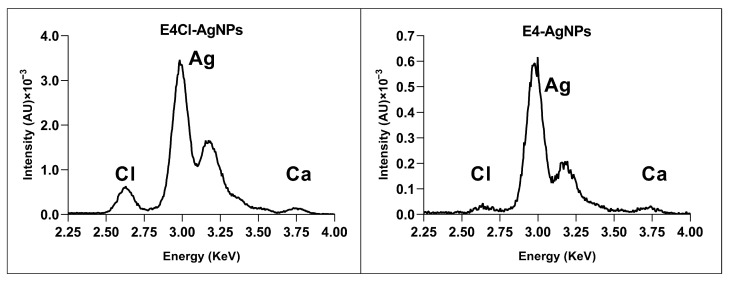
Representative TXRF analyses of the elemental composition of AgNPs. AU: arbitrary units.

TXRF analysis showed that silver was the main component of all the nanoparticles, appearing as two characteristic peaks in the 3–3.25 KeV region (Figure 5 and Figure S1). Traces of other elements in low concentrations appeared, showing a peak at 2.6 KeV, corresponding to chlorine. This element was found in a higher proportion in AgNPs prepared with broth containing it, but, in those without any added Cl^−^, only negligible amounts appeared (Figure 5 and Figure S1), probably coming from the contaminant traces of the reagents used to prepare the medium.

##### Crystallinity by Powder X-ray Diffraction (XRD)

**Figure 6:**
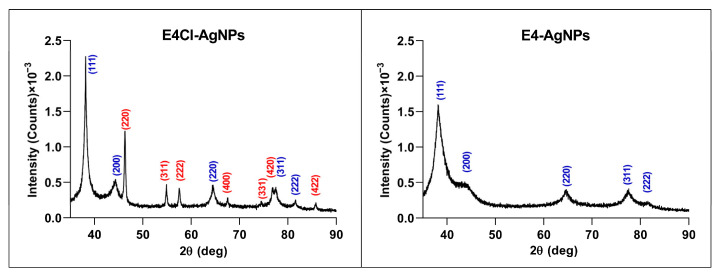
Representative XRD patterns of the AgNPs. Crystals’ planes are indicated for Ag^0^ in blue and AgCl in red.

XRD analysis showed peaks with 2θ values around 38.18, 44.34, 64.56, 77.50, and 81.50 degrees (Figure 6 and Figure S2). These values correspond, respectively, to the (111), (200), (220), (311), and (222) planes of the face-centered cubic structure of metallic silver crystals (Joint Committee on Powder Diffraction Standards (JCPDS) file 04-0783). Nevertheless, in the AgNPs from BG-11 broths (with Cl^−^), additional peaks were observed with 2θ values of 46.26, 54.88, 57.48, 67.48, 74.50, 76.80, and 85.80 degrees, corresponding, respectively, to the planes (220), (311), (222), (400), (331), (420), and (422) of the face-centered cubic structure of AgCl crystals (JCPDS file 31-1238). In the AgNP samples from BG-11A, the peaks associated with AgCl were not detected. These results show the presence of small amounts of AgCl with the more abundant Ag^0^ crystals in the AgNPs produced from the Cl^−^-containing broths.

##### AgNP Core Size and Shape by Transmission Electron Microscopy (TEM)

**Figure 7:**
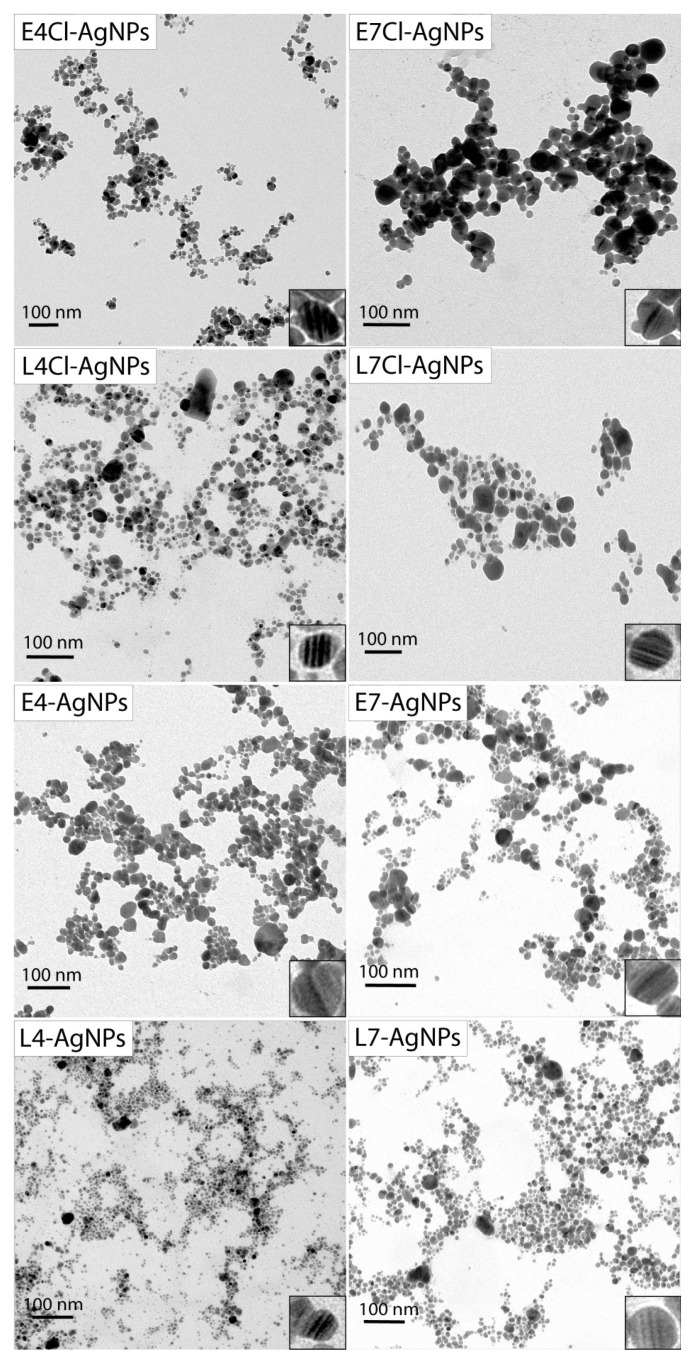
TEM representative images of the AgNPs. Lower-right corner insets in each panel show an amplified image of a nanoparticle with internal parallel patterns.

The analysis of representative TEM images allowed us to determine the shape and size of the AgNPs. The nanoparticles analyzed in this study presented a quasi-spherical shape, with a minority showing other morphologies (Figure 7). The TEM images also registered different levels of AgNP aggregation, with the most aggregated being the E7Cl-AgNPs and the L7Cl-AgNPs, while the less aggregated ones were the L4-AgNPs and the L7-AgNPs. Some nanoparticles presented internal structures with zones of differentiated density, appearing as alternated darker lines inside the nanoparticles.

**Figure 8:**
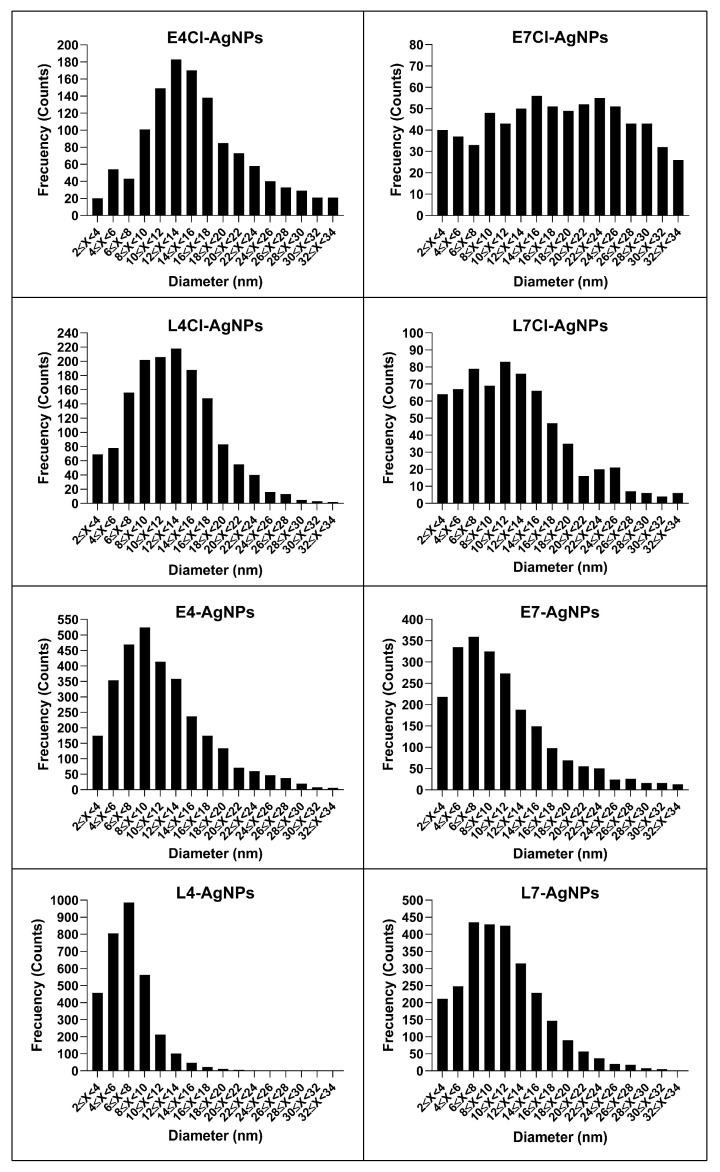
Distribution of AgNPs’ core diameters.

The size distributions of the AgNPs were different depending on the AgNP type (Figure 8), and the average diameter of the AgNPs’ cores was in the range of 7–21 nm, with some differences depending on the phase of the culture and the presence or absence of Cl^-^ in the media (Table 1). Thus, the nanoparticles produced from broths of early-phase cultures were bulkier than their counterparts from late-phase ones. Moreover, the size of the AgNPs seemed to vary depending on the presence or absence of Cl^−^. The AgNP sizes were around 7–11 nm and 12–21 nm for broths from cultures without Cl^−^ or with it, respectively.

The polydispersity index (PDI) was lower than 0.40, from 0.21 to 0.34 for nanoparticles produced with broth from pH 4 media, thus indicating moderate dispersity, according to dispersity scales previously proposed [64]. In contrast, the corresponding AgNPs prepared with pH 7 broths had a higher PDI, between 0.41 and 0.58, except for the L7-AgNPs (PDI of 0.32). The histograms of the distribution of AgNP sizes clearly show these polydispersity differences (Figure 8).

##### The Z-Potential and the Hydrodynamic Diameter of the AgNPs by Dynamic Light Scattering (DLS)

The AgNPs showed negative Z-potentials from −22 to −37 mV and hydrodynamic diameters varying from around 64 to 128 nm, depending on the AgNP type (Table 1). In general, the nanoparticles from late-phase cultures had consistently lower absolute values of the Z-potential and smaller sizes than the corresponding ones from early-phase broths, and these differences were larger among those prepared from media with Cl^−^ than when considering nanoparticles from media without it.

The PDI data obtained from the DLS analyses indicated relatively low dispersity in most cases (<0.4), except for L4Cl-AgNPs, E4-AgNPs, and E7Cl-AgNPs.

The ratio of the hydrodynamic to core diameter varied from about 4.5 to 12 depending on the type of AgNP, with L4-AgNPs showing the largest and L7Cl-AgNPs the smallest. The thickness of the corona, calculated as the difference between the hydrodynamic and core average ratio, ranged from 25 to 54 nm, with the L7Cl-AgNPs and the E7Cl-AgNPs having the thinnest and the thickest ones, respectively.

##### Corona Composition by Fourier Transform Infrared Spectroscopy (FTIR)

**Figure 9:**
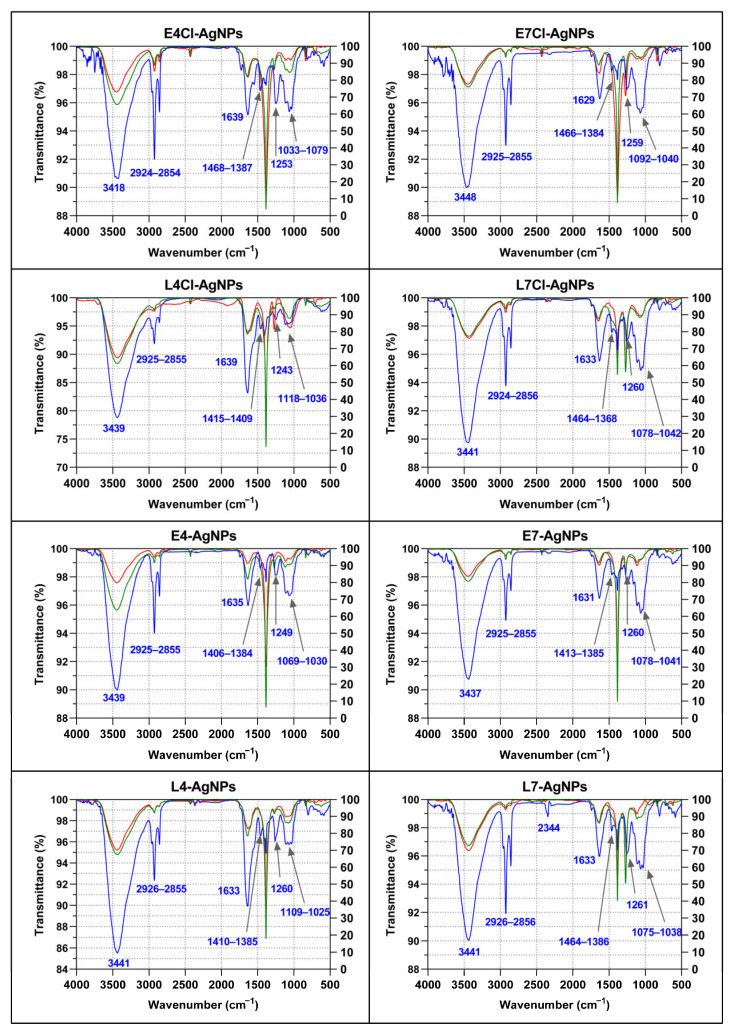
Characterization of the corona components of the AgNPs and the broths by FTIR. AgNPs’ spectra are shown in blue, the broths before the synthesis in green, and the broths after the synthesis in red.

All the AgNPs presented a wide peak in the 3430–3440 cm^−1^ region, a triplet signal at around 2854–2956 cm^−1^, a peak at around 1629–1639 cm^−1^, three subregions at the fingerprint region corresponding to 1384–1468 cm^−1^, a single peak at 1243–1261 cm^−1^, and a triplet at about 1030–1118 cm^−1^ (Figure 9). The FTIR spectra of the broths before and after synthesis showed very little difference.

All the spectra showed peaks at about the same wavenumbers, but their relative transmittances were different in some cases, mainly within the fingerprint region. Lower transmittance of the band at about 1640 cm^−1^ compared to the triplet around 1060 cm^−1^ was observed in AgNPs from pH 7 broths compared to those from pH 4 broths. This difference was especially noticeable for AgNPs from late-phase cultures, with L4Cl-AgNPs and L4-AgNPs presenting lower transmittance at 1639 cm^−1^ or 1633 cm^−1^, respectively, in contrast to the other AgNPs.

Following authors who have studied FTIR peak assignment to biomolecules [65], we could tentatively consider that the differences in the composition of the AgNPs were related to proteins, carbohydrates, and aliphatic chains containing compounds, which may have differed in proportion depending on the AgNP type. Thus, the AgNPs prepared from pH 4 broths seemed to have a higher proportion of proteins (peak around 1640 cm^−1^, usually ascribed to these macromolecules) to carbohydrates (signals at around 1060 cm^−1^) than the AgNPs from pH 7 broths. This ratio was much higher in the AgNPs from late-phase cultures, independently of the Cl^−^ presence in the broths. No peak at the region of 1710–1760 cm^−1^, correlated with lipids [65], was observed in these spectra. Signals appearing at 2854–2956 cm^−1^, usually assigned to aliphatic chains, were detected, in most cases showing higher intensity than the carbohydrate peaks or than the protein peaks in some cases. The aliphatic chain peaks’ intensities depended on the AgNP type, with the highest produced with AgNPs from pH 4 early-phase broths. These results indicate a different composition of biomolecules in the corona of the AgNPs depending on the culture media pH and the growth phase of the cultures used for their synthesis. No significant difference arose concerning the presence or absence of Cl^−^ in the broths.

Some differences observed among the broth’s spectra depended on the cultures from which they proceeded. Thus, a strong peak at about 1260 cm^−1^ observed in the broths from late-phase cultures at pH 7 did not appear in the other broths. Differences between the corresponding broths before and after the AgNPs’ synthesis were not relevant, except for a decrease in the peak at 3418 cm^−1^ and at the region of 1033–1079 cm^−1^ in the E4Cl-AgNP spectrum, and mainly at 3439 cm^−1^ for the E4-AgNP ones.

#### 2.3.2. Biological Activity of the AgNPs

##### Antibacterial and Antibiofilm Activity

The use of the microdilution method allowed us to evaluate quantitatively the growth of three Gram-negative (*E. coli*, *Klebsiella pneumoniae*, and *Pseudomonas aeruginosa*) and Gram-positive bacteria (*Bacillus subtilis*, *S. aureus* and *Staphylococcus epidermidis*) (see Section 4) in the presence of several concentrations of the different AgNPs, to calculate the antibacterial parameters: minimal inhibitory concentration (MIC), minimal bactericidal concentration (MBC), inhibitory concentration of 50% growth (IC_50_), and inhibitory concentration of 50% biofilm formation (ICb_50_) (Table 2). These studies included the antibiotic streptomycin (Sm) and silver nitrate (${AgNO}_{3}$) as controls.

The MIC values for most of the AgNPs were lower than for Sm for almost all the test bacteria, indicating higher activity related to the weight of Ag in the AgNPs or to the antibiotic one. Exceptions were for E4-AgNPs, E7-AgNPs, and L7-AgNPs, which were less active than the antibiotic against *K. pneumoniae*. Comparisons of the MIC values for Sm against all the tested bacteria showed significant activity against this species. On the contrary, in the case of *S. epidermidis*, a Sm-resistant phenotype was found, but not for the AgNPs, which, in general, were quite active against this species, the most susceptible to the AgNPs among the tested Gram-positive bacteria.

In general, the AgNPs’ MICs were higher than those of ${AgNO}_{3}$, with some exceptions in which they were similar—for instance, in the L4Cl-AgNPs. In some cases, such as E7-AgNPs, the difference was more than ten times higher than for silver nitrate. Most AgNPs were thus less active than silver nitrate.

The MIC and the corresponding MBC values were the same for most AgNPs, indicating bactericidal behavior. However, some AgNPs showed a higher MBC against several bacteria, particularly E7-AgNPs against Gram-negative bacteria except *E. coli*, and against Gram-positive bacteria except *B. subtilis*; L7-AgNPs against *K. pneumoniae*, *S. epidermidis*, and *B. subtilis*; L4-AgNPs against *P. aeruginosa*, *S. aureus*, and *S. epidermidis*; L4Cl-AgNPs against *P. aeruginosa* and all the Gram-positive bacteria; E4Cl-AgNPs against *S. epidermidis*; E4-AgNPs against *P. aeruginosa*, *S. epidermidis*, and *B. subtilis*; L7Cl-AgNPs against *S. aureus* and *S. epidermidis*; and E7Cl-AgNPs against *S. epidermidis*. This higher MBC compared to the MIC is particularly common against *S. epidermidis* for all the nanoparticles showing this behavior. However, in some cases, the MBC/MIC ratio was higher than 4, indicating bacteriostatic behavior [66], such as for L7-AgNPs, L4Cl-AgNPs, and L4-AgNPs against *S. epidermidis* and the last one against *P. aeruginosa*. Silver nitrate also had a high MBC/MIC value against the two *Staphylococcus* species tested and against *S. epidermidis*, in the limit of indicating a bacteriostatic mechanism (MBC/MIC = 4).

The MICs and MBCs indicated that AgNPs produced from cultures with chlorine are mostly more efficient antibacterial agents (MICs ≤ 5 µg/mL) than those from broths without it. The nanoparticles produced from pH 7 cultures with chlorine stand out especially, with an MIC range of 0.84–3.47 µg/mL, while similar nanoparticles synthesized without chlorine ranged from 2.63 to 23.13 µg/mL. AgNPs from pH 4 media showed a similar effect, with MICs of 0.47–5 µg/mL versus 0.92–14.74 µg/mL, with or without chloride, respectively. AgNPs from pH 4 media were generally more active than those from pH 7 ones, with lower differences dependent on chlorine.

The activity against any of the tested bacteria was higher for the late-phase nanoparticles than for the corresponding ones from the early phase, except for the L4- and E4-AgNPs against *S. aureus* and *B. subtilis*, for which the opposite was observed, and for the L7Cl- and E7Cl-AgNPs, which showed very similar MICs against all the bacteria except *E. coli*.

The most susceptible to the AgNPs among the Gram-negative bacteria was, in general, *P. aeruginosa*, and the activity against three different strains of this species confirmed that this high susceptibility was mostly strain-independent (Table S1).

The most active AgNPs against any of the test bacteria were L4Cl-AgNPs, with MIC values in the range of 0.47–1.87 µg/mL, while the least active were E7-AgNPs, with MICs ranging from 5.78 to 23.13 µg/mL.

The antibiofilm activity of the AgNPs was evaluated by using the ICb_50_ parameter. The ICb_50_s in several cases were quite similar to the corresponding IC_50_s; however, a tendency for higher values of ICb_50_ than IC_50_ was observed, indicating that inhibiting biofilm formation requires a higher concentration of AgNPs than inhibiting planktonic cells’ growth. Moreover, in a few cases, the ICb_50_ values doubled the IC_50_ ones, but, in most cases, the differences did not reach these levels. For silver nitrate, a similar effect could also be observed, but, for the cases of *P. aeruginosa* and *S. epidermidis*, the ICb_50_ values were much higher than the IC_50_ values, with not much difference between the corresponding values for the AgNPs.

##### Antibacterial Activity of AgNPs Harvested at Different Times in the Synthesis Reaction

The evaluation of the antibacterial activity against *E. coli* and *S. aureus* of L4Cl-AgNPs and L4-AgNPs harvested after different reaction times (Table 3) showed that the AgNPs from the earlier harvests were the most efficient. In the case of the L4Cl-AgNPs, which usually are collected after 72 h when maximum production occurs, the highest activity corresponded to the 24 h harvest batch. For L4-AgNP synthesis, in which the maximum production is at 200 h, the batch obtained after 100 h of synthesis showed the highest activity. When comparing the activity results with the structural changes observed in the UV–Vis spectra (Figure 3), a relationship arose with the most stable AgNPs, corresponding to later harvests, showing lower activity for the two model AgNPs tested.

##### Antibacterial Activity of Successive Batches of AgNPs Using the Same Broth

The results of the antibacterial activity testing against *E. coli* and *S. aureus* of the successive AgNP preparations (Table 4) showed decreasing activity of the consecutive batches, except for the second ones, which were very similar to those of the former ones. However, the slower synthesis and lower yields observed for the third and fourth syntheses (Figure 4) led to lower, but still satisfactory, activity of the produced AgNPs. The IC_50_ and ICb_50_ parameters behaved similarly.

##### AgNPs’ Cytotoxicity on Cultured Intestinal Human Cells

To use the AgNPs as antibacterial agents, it is necessary to ensure that they are not cytotoxic to eukaryotic cells. We used the three in vitro assays described in the Section 4 to test our AgNPs. The results showed different levels of toxicity of the several AgNPs (Figure S3), as reflected by the concentration reducing cells’ viability by 30% (IC_30_) (Table 5). The most cytotoxic AgNPs were three of the AgNPs synthesized with broths with Cl^−^, L4Cl-AgNPs, followed by E7CL-AgNPs and L7Cl-AgNPs, while the least cytotoxic were the ones prepared from broths from cultures at pH 4 without Cl^-^ from any of the two growth phases. E4Cl-, E7-, and L7-AgNPs produced intermediate toxicity. The three endpoints evaluated generally presented comparable results, although the plasma membrane integrity showed the highest sensitivity and lysosomal physiology with NRU the least.

Besides the cytotoxic profile of the AgNPs, the most relevant parameter to determine the possible usefulness of the AgNPs would be the level of viable cells when using the concentration of AgNPs corresponding to the MICs for each test bacteria (Table 2). As can be observed, for most AgNPs, the percentage of viable cells at the MICs was higher than 70%, indicating that the use of concentrations of AgNPs producing the complete inhibition of the test bacteria growth showed little or no damage to the human cells (Table 2). Nevertheless, in some cases, the viable cells at the MICs were lower than 70% or even lower than 50%.

#### 2.3.3. Antibacterial Synergy of the AgNPs with Classic Antibiotics by the Checkboard Assay

The measurement of the antibacterial activity of combinations of AgNPs with one out of seven classical antibiotics against *E. coli* and *S. aureus*, and the calculation of the fractional inhibitory concentration index (FICI) [67,68,69] and the modulatory factor (MF) [49], showed synergic behavior in some cases (Table 6). Strong synergy of the AgNPs was found with Sm or kanamycin (Km) against both tested bacteria (FICI ≤ 0.5; MF = 16–32). Moreover, almost all AgNPs showed a similar positive synergic behavior with ampicillin (Ap) against *S. aureus* and tetracycline (Tc) against *E. coli* (FICI ≤ 0.5; MF = 2–8). The effects of most AgNPs with Ap or ertapenem (Ep) were additive against *E. coli* and with Tc against *S. aureus* (0.5 < FICI ≤ 1; MF = 2–8). Furthermore, combinations of certain AgNPs with nalidixic acid (Nx) or ciprofloxacin (Cp) showed less regularly distributed additive results, depending on the AgNPs and the bacteria. Moreover, some AgNPs produced indifferent effects with Ap, Ep, Cp, Nx, and Tc (1 < FICI ≤ 2; MF = 1) and no combination generated antagonism (FICI ≤ 4). Using a stricter interpretation of the FICI [68], positive synergic activity can be considered for all AgNPs with Sm or Km against both bacteria, with Ap against *S. aureus* (except for the E7-AgNPs) and with Tc against *E. coli* (FICI ≤ 0.5; MF = 2–8). Despite using the strictest criterion for synergy estimations, FICI ≤ 0.5, the FICI values ranged, depending on the considered AgNPs, from 0.250 to 0.5 for Ap and from 0.188 to 0.5 for Tc, while, for Sm against *E. coli*, the range was 0.047–0.313, that against *S. aureus* was 0.063–0.188, and that for Km was 0.063–0.156 and 0.125–0.375, respectively, against the same bacteria. Thus, these results indicate the net specificity of the synergy with Ap against *S. aureus* and Tc against *E. coli*. Moreover, there were differences in the synergy levels against the two bacteria in combination, showing a synergy against both, as in the case of Km and Sm. Thus, in general, the synergy of the AgNPs with these two antibiotics was higher against *E. coli*, except when, for some AgNPs, the synergy with Sm was higher against *S. aureus*.

Differences in synergy levels also existed depending on the AgNP type. For instance, among the nanoparticles showing synergic behavior with Ap (against *S. aureus*), the lowest value of the FICI, and thus the highest level of synergy, was observed for the E7Cl-AgNPs. Among those nanoparticles synergic with Tc (against *E. coli*), the L7-AgNPs produced the best effect, followed by the E7Cl-AgNPs. L4-AgNPs and L7Cl-AgNPs generated the maximum synergy with Km against *E. coli*, but, against *S. aureus*, three AgNPs showed good synergy, while the E7-AgNPs produced much lower levels. Finally, the L4-AgNPs gave the maximum synergy with Sm against *S. aureus*, but, for *E. coli*, those generating a higher effect were the L7Cl-AgNPs, followed by E4Cl-AgNPs. While the synergy levels with Km were, in general, for most AgNPs, higher on *E. coli* than on *S. aureus*, this correlation was not so clear for Sm depending on the AgNPs; four of these showed a greater effect on *E. coli*, but the others showed an equal or lower effect than against *S. aureus*. Overall, the highest synergic effect was observed for L7Cl-AgNPs with Sm against *E. coli*, while the lowest was for L4-AgNPs, L4Cl-AgNPs, and E7-AgNPs with Tc against *E. coli*, and L4-AgNPs and L7-AgNPs with Ap against *S. aureus*. Thus, the best combinations for good synergy would be Sm (or Km) + L7Cl-AgNPs and Tc + L7-AgNPs against *E. coli*, and Ap + E7Cl-AgNPs, Sm (or Km) + L4-AgNPs, and Km + E7Cl-AgNPs (or L7-AgNPs) against *S. aureus*. From these results, we can deduce that AgNPs from cultures at pH 7 are better at producing a synergy with the tested antibiotics, in some cases for AgNPs from the late-phase broths and in some others from the early-phase ones, with some from media with chloride and some without it. Only AgNPs obtained with broths without Cl^-^ from the late phase of pH 4 cultures acted synergistically, and only against *S. aureus* and with the aminoglycoside antibiotics. Against *E. coli*, only AgNPs from the late phase of pH 7 cultures produced a synergy, while AgNPs from the early phase of pH 7 cultures containing Cl^−^ were better against *S. aureus*.

##### Insights into the Mechanism of the AgNPs’ Antibiotic Synergy

**Figure 10:**
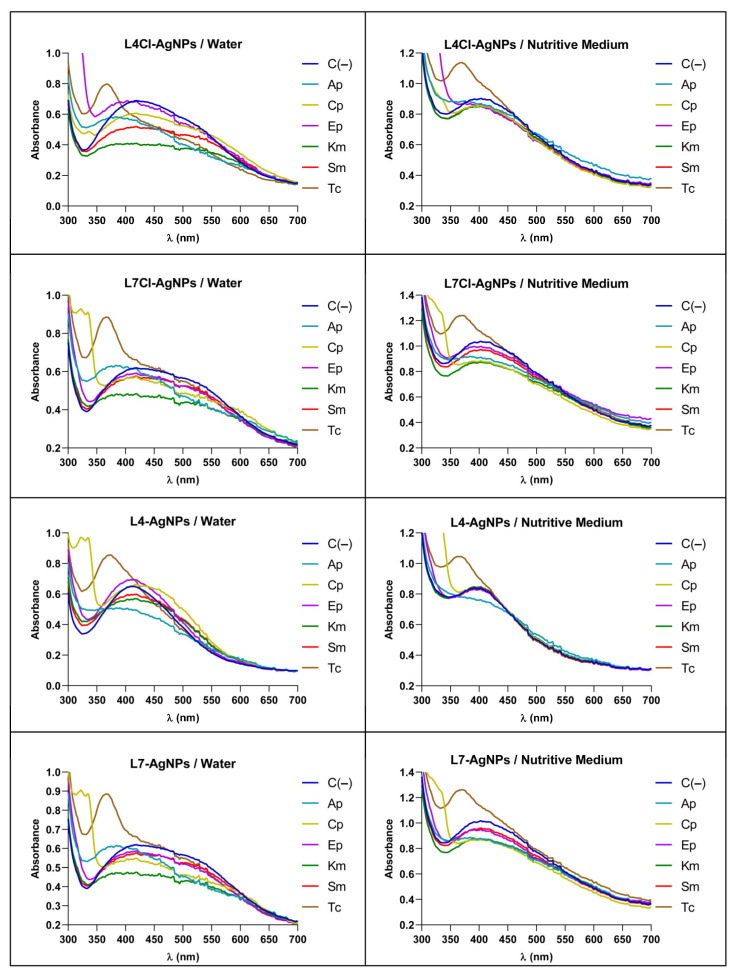
Effect of the presence of the antibiotics on the UV–Vis spectra of the AgNPs.

We studied the changes in the AgNPs’ UV–Vis spectra when mixed with the antibiotics to obtain some insights into the synergy mechanism. The AgNPs used as models were those generated with the late-phase culture broths. Changes observed in the spectra depended on the media in which both substances were combined, i.e., water or a nutritive medium (Figure 10). The spectra of all the tested AgNPs and antibiotics showed an absorbance decrease at λ_max_ when mixed in water or a nutritive medium. However, in water mixes, an additional increase in the absorbance at higher wavelengths appeared, indicating the aggregation of the nanoparticles. This increase did not appear in the nutritive medium mixes.

**Figure 11:**
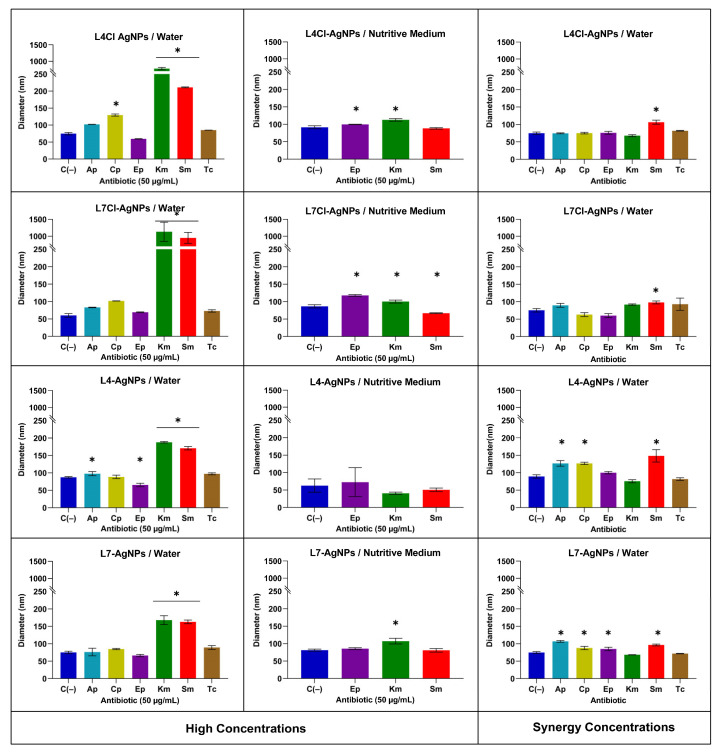
The hydrodynamic diameters of AgNPs in the presence of antibiotics. C(−) negative control, without antibiotic. (*) indicates a significant difference between the AgNPs’ sizes in the mixes with antibiotic and the C(−). *p*-value < 0.05.

Furthermore, we performed a study by DLS of the AgNP sizes in their mixes with antibiotics at the same concentrations used in the spectrophotometric analysis and in lower concentrations corresponding to values for which antibacterial synergy had been observed (Figure 11). The results at high concentrations showed an increase in the AgNP sizes, mainly for the antibiotics Sm and Km, when using water as the solvent, but almost no size changes from the AgNP controls without antibiotics in the nutritive medium. Moreover, in the mixes at low concentrations corresponding to a positive synergy, no difference in AgNP sizes was apparent.

### 2.4. ROS Production and Bacterial Killing by the AgNPs

**Figure 12:**
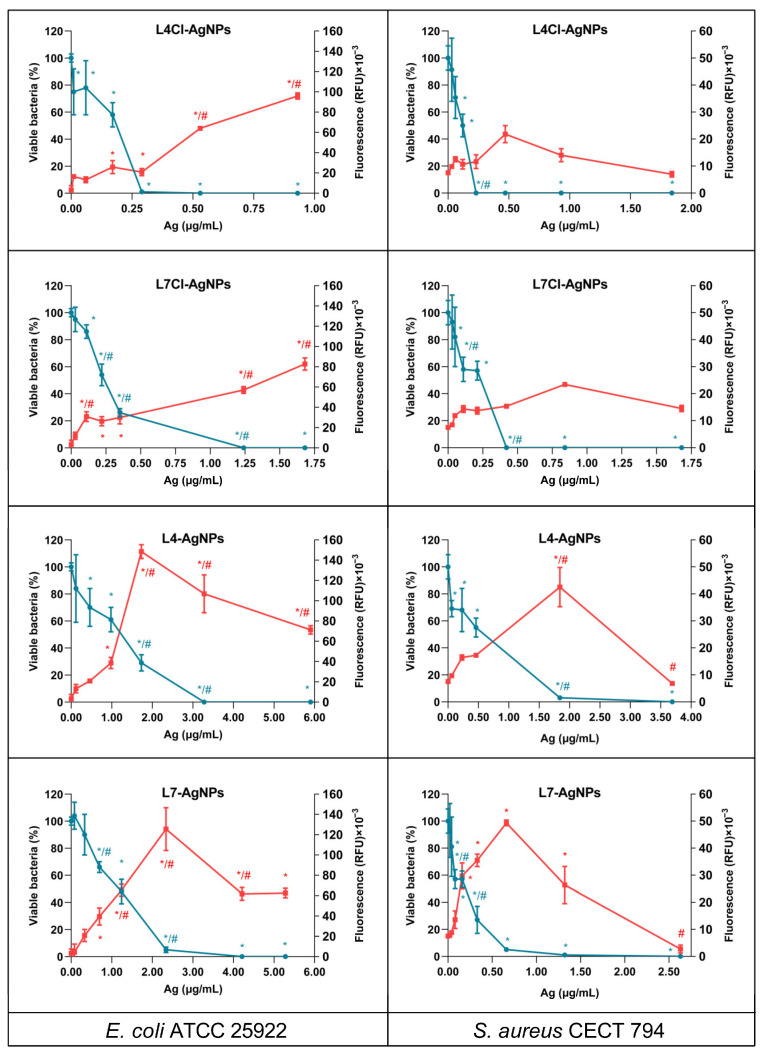
ROS production and viable cells after treatment with AgNPs. Viable cells % are shown in blue and fluorescence is in red. Statistically significant differences of each value with the untreated control (*) and with the immediately lower AgNPs’ concentration value (#) are indicated.

*E. coli* and *S. aureus* produced ROS accumulation over time when treated with different concentrations of the AgNPs from late-phase broths, the most active ones obtained in this work (Figure S4). After 4 h of treatment, ROS accumulation was not saturated, with different levels of ROS observed depending on the bacteria and the AgNP tested (Figure 12). The ROS accumulation was generally higher in *E. coli* than in *S. aureus*. Moreover, the assessment of the viable cells remaining after the 4 h of treatment showed that at the concentrations of the AgNPs producing maximum levels of ROS, low or null levels of viable cells were present. At concentrations of AgNPs lower than these, the viable cells decreased with the increasing concentrations of the AgNPs, but a correlation between the decrease in viability and the increase in ROS only appeared for AgNPs prepared using broths without Cl^−^. For the others, the viability decrease emerged at AgNP concentrations for which ROS accumulation was still low.

## 3. Discussion

This paper reports the synthesis of AgNPs using eight culture broths of a microalga isolated from the Tinto River, an acidic and microalgae-rich environment [70]. The use of culture broths from microalgae for the production of AgNPs is uncommon in the literature, since mainly extracts or cells have been previously used [30,33,34,35,37]. Using broths instead of extracts or cells simplifies the process and reduces the costs of AgNP production.

### 3.1. Microalga Growth and Tested Conditions

In this work, we tested the possibility of using a microalga to obtain culture broths for the green synthesis of AgNPs. The media used for microalga cultures were the general BG-11 medium and BG-11A, a BG-11-based medium containing no chloride, with two versions of each, adjusted to pHs 4 or 7. The microalga was able to grow in the four tested conditions. Since the microalga was previously isolated from an acidic habitat and grew at both tested pHs, we estimated it to be an acid-tolerant organism. Growth was faster at pH 7 than at pH 4, but the final pH of all the broths when collected was pH 9. However, we estimated that the synthesis and the characteristics of the AgNPs produced using cultures in media with one or the other pH would be different, since there was a significative difference in the growth curves depending on the original pH of the medium. No relevant growth differences were observed depending on the chloride’s presence. However, the presence or absence of chloride might affect the composition of the broth during microalga growth. Moreover, the presence of Cl^−^ may affect different aspects of the AgNPs’ synthesis and their physicochemical and biological activity and properties, as previously described [61,62,63]. Additionally, the growth phase of the cultures from which the broths proceeded could affect the composition and perhaps the kinetics of AgNP synthesis and the properties of those produced, as reported in some previous studies for different metallic nanoparticles [63,71,72,73,74,75,76]. In the present work, the three parameters changed to obtain the broths, namely the pH, Cl^−^ presence, and growth phase of the cultures, affected the kinetics and characteristics of the AgNPs synthesized with them in different ways.

### 3.2. Kinetics of AgNP Synthesis

The synthesis of AgNPs followed spectroscopically showed that the spectra observed had λ_max_ in the range usually found for AgNPs [25], albeit with differences depending on the nanoparticle type, indicating differences in their physicochemical characteristics. The kinetics of each AgNP reached a plateau at different incubation times, but further incubation led to a decrease in absorbance at λ_max_ and signs of aggregation, such as an increase in absorbance at higher wavelengths. This behavior is common in previous reports of biogenic AgNPs [63,77].

The synthesis of each type of AgNP showed kinetics with different synthesis rates, which seems to be related to the characteristics of the broth used. Reactions were faster for broths containing Cl^−^, when the pH of the medium used for microalga culture was acidic, or when using broths from the early-phase cultures.

Nanoparticles from the reactions with broths containing Cl^−^ showed some concentration of this element by TXRF, and AgCl crystals appeared in their XRD analyses that did not in the AgNPs prepared from non-Cl^−^-containing broths.

It is unknown whether the higher synthesis rates observed when using broths containing Cl^−^ were due to the presence of Cl^−^ itself or to the different compositions of the broths from the microalga grown in its presence. However, the lack of relevant differences in the growth curves of the microalga in media with and without Cl^−^, and the minor difference in the corresponding FTIR spectra, may suggest, in this case, that the broth composition was not so different and relevant as the presence or not of Cl^−^. In previous works by our group, a similar effect on the AgNP production rates was observed for the synthesis of AgNPs using *Pseudomonas alloputida* culture broths [63], but a decrease in the rate and yields of AgNP synthesis in the presence of Cl^−^, especially at 4 °C, was found when using broths from psychrophilic bacteria cultures [62]. The addition of Cl^−^ salts to AgNP preparations modifies their aggregation behavior in a way suggesting that the Ag^+^ released from the AgNPs could be immediately precipitated on the surfaces of the nanoparticles as AgCl, making them grow [61]. Some authors have reported that the presence of Cl^−^ may produce AgCl crystals, due to the low solubility of this compound, leading to the formation of nanoparticles containing them [63], which could facilitate nanoparticle synthesis. The AgNPs reported here were composed mainly of Ag^0^, perhaps because of the low concentration of Cl^−^ in the broths, but several cases in the literature report different proportions of AgCl to Ag^0^ in AgNPs prepared in media containing different amounts of Cl^−^ in a way not directly related to the Cl^−^ concentration. Thus, for example, using nutritive broth from a *Pseudomonas* sp. THG-LS1.4 culture produced AgNPs with no detected AgCl crystals [78], even though the medium contained NaCl. However, using *Pseudomonas alloputida* B003 UAM culture broths in the same medium led to AgNPs with small amounts of AgCl crystals [63]. In addition, AgCl crystals predominated in AgNPs produced by other researchers from bacterial cultures in the same medium [79] or using a different one with the same concentration of NaCl [80]. All this would indicate no direct relationship between the Cl^−^ concentration and the presence of AgCl crystals in the nanoparticles, as discussed in a previous report [63]. The organic components of the broths from cultured organisms might affect the production of AgCl nanoparticles, perhaps depending on the concentrations of reductant and protector compounds or the ratio between them. It seems that a complex effect of the presence of Cl^−^ on the rate of synthesis and the formation of AgCl crystals in the final AgNPs, also depending on other conditions such as the temperature [62] and the composition of the biological material used in the synthesis, may exist.

Some authors have reported the effects of the pH on AgNP synthesis [81,82]. However, in our case, the pH of the broths after microalga growth had changed to pH 9. The microalga grew differently depending on the original pH of the culture medium, which could have produced different broth compositions. Thus, differences in the reaction rates when using broths from cultures originally set to pH 4 or 7 would have to be related to the different broth compositions. The analysis of the FTIR spectra of broths from cultures set at pH 4 or 7 showed some differences between the corresponding ones, albeit mainly for broths from late-phase cultures for which some clearly different peaks were observed (mostly the peak around 1260 cm^−1^). Nevertheless, this peak was not seen in the spectra of the corresponding AgNPs. Besides this, syntheses were faster when using broths from cultures initiated at pH 4 than those from cultures started at a neutral pH.

The microorganism growth phase from which the broths proceeded affected the process of AgNP production. Thus, broths from the early growth phase produced faster syntheses. Similarly, a previous study showed that broths from the exponential growth phase of a bacterial culture synthesized AgNPs faster than those from stationary ones [63]. However, in other reported cases, the late [72] or early stationary phases [74] of the microorganism cultures were better at producing AgNPs. Moreover, in another case, the synthesis was independent of the growth phase [71]. These studies thus indicate that the AgNP production rates may or not be dependent on the growth phase, or, when they are dependent, the late or the early growth phases could be more suitable for the production of AgNPs, depending on the microorganism and the protocol used.

### 3.3. Insight into the AgNPs’ Synthesis over Time and Reuse of Broths

To obtain insights into the AgNP synthesis process, we harvested AgNPs during their synthesis by taking samples of the reaction mix at different times up to the kinetics plateau. We used two types of AgNPs as models, L4Cl-AgNPs and L4-AgNPs, which showed a notable difference in the time needed for the reaction to reach the plateau. After harvesting the AgNPs from the reaction mix and washing them, the spectra of the AgNPs with faster kinetics, L4Cl-AgNPs, indicated instability inversely related to the reaction time. Moreover, the antibacterial activity of these AgNPs showed a decrease, also depending on their synthesis time. From this, we concluded that an earlier harvest produces lower yields of less stable and more active nanoparticles. The L4-AgNPs, with slower synthesis kinetics, showed an increase in stability with longer synthesis times, and they sedimented poorly during their collection from the reaction mix by centrifugation as well. The difficulties in sedimentation may be due to an increase in the corona size, composed of lighter-than-the-core compounds, making the AgNPs more reluctant to settle and more stable. These results indicate that, in the initial phases of synthesis, the AgNPs are less stable, so we can speculate that, in a fast synthesis, the AgNPs do not have enough time to establish a proper corona to protect themselves from the aggregation observed during their washing, mainly at short reaction times. Moreover, for AgNPs whose synthesis is slower, the cores have sufficient time to acquire the necessary capping materials and are more stable, even in a short reaction time. In this last case, at longer reaction times, the amount of material around the cores can increase greatly, producing AgNPs with larger coronas, in agreement with the DLS results, which would make their sedimentation difficult. From the point of view of process optimization, obtaining AgNPs at shorter reaction times produces more active materials, but higher yields could be obtained by sacrificing some activity at longer synthesis times. Since the differences in the antibacterial activity among AgNPs of the same type but harvested at different reaction times are not large, and they are more stable at longer times with higher yields, the collection of the AgNPs at the reaction time of maximum production may be advantageous. The dependence of the AgNPs’ antibacterial activity on the harvest time should be considered, as well as their final yields and stability, in subsequent trials or for industrial production.

Reusing the broths from the synthesis of an AgNP batch in other consecutive syntheses would be of interest in reducing production costs. We prepared four consecutive AgNP batches using the same broth and tested their antibacterial activity. The second round of synthesis produced AgNPs without significant changes in the kinetics of the process, the spectra of the resulting AgNPs, or their antibacterial activity. However, the third and fourth rounds showed lower synthesis rates, yields, and antibacterial activity. Similarly, bacterial broths used for the green synthesis of other AgNPs [63] produced AgNPs in a second synthesis round without deleterious consequences for the synthesis rates and the antibacterial activity of the AgNPs. The concentration of compounds needed for Ag^+^ reduction would have decreased significantly after the second round of synthesis, which may be a limiting factor in the process. Some studies have shown that the concentration ratio of the Ag^+^ precursor/biological material affects the characteristics of the AgNPs obtained [77], so a similar effect could have occurred in the successive syntheses tested here. In our experiment, this ratio increased in each round of synthesis, because reusing the broths implied a decrease in the available compounds as some of these had to be oxidized to reduce Ag^+^ and others attached to the AgNPs’ cores to build the corona, while ${AgNO}_{3}$ was added each time at the same concentration. Whether or not this is a general finding should be tested by analyzing other cases because of its probable relevance in reducing production costs. To the best of our knowledge, no other researchers have yet addressed this possibility.

### 3.4. Physicochemical Characteristics of the AgNPs

Differences in the physicochemical characteristics among the eight types of AgNPs obtained in this work were apparent in terms of their size, dispersity, Ag^0^ or AgCl content, and corona composition.

Concerning the AgNPs’ core shape, size, and dispersity, the AgNPs obtained were of the same quasi-spherical shape, with little difference in size, mainly concerning the presence of Cl^−^, which produced the largest AgNPs. The early-phase broths also generated larger AgNPs than the late-phase ones. The measured AgNP sizes were small compared to those reported in the literature [24,25,26,27,83]. The λ_max_ values of the AgNPs’ UV–Vis spectra are related to the size and dispersity of the nanoparticles [58,63], with narrower spectral peaks and a lower λ_max_, usually associated with less size dispersion and smaller nanoparticle cores, respectively. In our case, such a correlation appeared when considering the parameters assessed by TEM. The pH of the culture media used to set up the cultures to obtain broths for AgNPs’ synthesis seems to be the most differentiating parameter influencing the dispersity of the AgNPs cores reported here, since the AgNPs from pH 4 media were moderately dispersed in most cases, while those obtained from pH 7 ones showed larger dispersity. When comparing the PDIs of the hydrodynamic diameters and those corresponding to the core diameters, it seems that the corona formation led to the homogenization of the PDI values, which were more similar for the AgNPs’ hydrodynamic sizes than for the AgNPs’ cores. Since the final pH of all the broths used for AgNP synthesis was the same, namely pH 9, these differences are likely due to the various compositions of them and not to the pH itself.

The analysis by DLS showed differences in hydrodynamic size and Z-potentials and the presence of coronas of different thicknesses around the cores. Differences in the Z-potential seem to be related to the growth phase of the broths used to prepare the AgNPs, with differences increased in the AgNPs with Cl^−^. Some authors consider the Z-potential as indicative of the AgNPs’ stability, with those having higher values being more stable due to the stronger repulsion among the nanoparticles with the same charge sign, but other reports do not agree [84]. All the AgNPs obtained in this work had Z-potentials in the range considered to indicate stability [84]. Besides this, negative Z-potential values seem to condition the antimicrobial activity of the nanoparticles by complicating the approach to the negatively charged bacterial envelopes [85]. However, in previous reports, this effect has not been detected [63], and it was not seen in this study either.

We speculated that the corona thickness, structure, and composition could influence the AgNPs’ activity, since Ag^+^ release or the interaction with cellular components could be affected. However, no correlation between the calculated corona thickness of our AgNPs and their antibacterial activity was apparent. Factors such as the structure and composition of the corona and their capability to interact with the bacterial cells may have affected the AgNPs’ antibacterial activity in an unknown way. In a previous work, our group showed that the biogenic AgNPs obtained conserved their UV–Vis spectra but showed a decrease in their antibacterial activity after ageing for several months, which may have been related to changes in the corona structure, rather than depending on the Ag core itself [62]. Techniques for the study of the corona structure and composition, which, in green synthesized AgNPs, are intrinsically complex, may be of great interest to clarify its role in the biological activity of AgNPs.

Many authors have used FTIR to evaluate the organic composition of the AgNPs’ corona, but the adscription of IR bands to specific molecules is somewhat speculative in the case of complex mixes, and, in most cases, adscription is limited to the types of biomolecules or their relative proportions [65]. In this respect, all AgNPs showed similar spectra with representative bands of the functional groups found in proteins, carbohydrates, and aliphatic compounds. Moreover, the spectra of the various AgNPs obtained in this work showed differences in the relative intensity of the peaks corresponding to these compounds, depending on the broths used for their synthesis, mainly on the growth phase and pH. A higher proportion of signals corresponding to proteins than those from carbohydrates was visible in the spectra of the AgNPs from cultures initiated at pH 4 than in those from pH 7 or for late-phase than early-phase broths. These results agree with the differences in the microalga growth curves at the two pHs and support that the differences observed between Cl^−^-containing and not-containing AgNPs were probably more related to the presence of this anion during the synthesis than to differences in the broth composition, which the FTIR spectra do not support.

The comparison of the AgNPs’ FTIR spectra and those from their corresponding broths indicated that the composition of the corona in each nanoparticle was different and that the compounds in them were selected from the compounds present in the broths but not in the same proportion; thus, for instance, the prominent peaks appearing in the broths at around 1384 cm^−1^ and 1272 cm^−1^ did not appear noticeably in the AgNPs’ spectra and seemed to have not been taken from the broths.

### 3.5. Antibacterial Activity

The formation of biofilm structures by bacteria protects them from the action of antimicrobials, making the bacteria more difficult to eliminate [86,87]. For our AgNPs, in general, the ICb_50_s were low, not even doubling the corresponding IC_50_s. This suggested a small protective effect of the biofilm structures against the AgNPs, making them good antibiofilm agents, better than other compounds. Some other authors have shown that microbiologically synthesized nanoparticles can be good antibiofilm agents, as reviewed in [88].

By comparing the antibacterial activity of the AgNPs and ${AgNO}_{3}$, the higher activity of ionic silver than most of the AgNPs become clear, even though, in a few cases, the activity parameters were very close or, as in the case of L4Cl-AgNPs, even lower than for ${AgNO}_{3}$. In the literature, reports of the higher activity of AgNPs over silver ions or vice versa have been found [89,90]. In a study [91], silver ions displayed higher activity than AgNPs, but the reported ratio of the MIC values of Ag^+^ to AgNPs was small (twice or four times, depending on the type of AgNPs). However, in another report, they were much higher [92]. These different results indicate that any situation is possible depending on the AgNPs. In our case, the ratios were 2 or less for most AgNPs. Thus, different AgNPs could be better or worse antibacterial agents than silver ions, as in our study, even though, among our AgNPs, the situation of silver ions being more effective than AgNPs was more general, but with a low activity ratio.

In this study, we included the antibiotic streptomycin as a control for the susceptibility levels of the test bacteria in the same assays used to test the AgNPs. The MICs were noticeably higher for the antibiotic than the AgNPs, indicating that the latter were the more active antibacterial agents. Moreover, *S. epidermidis* resulted in being resistant to Sm, while all the AgNPs, at different levels, were quite effective. This situation is paradigmatic with AgNPs, but not a classic antibiotic, being able to control the growth of this bacterium, highlighting the possible utility of AgNPs against some antibiotic-resistant bacteria. Several studies have also claimed this use of AgNPs, for instance, among many others, against multi-drug-resistant clinical bacterial isolates [93] or against multi-resistant strains of some of the most dangerous bacterial species, such as *Acinetobacter baumannii*, *P. aeruginosa*, and *S. aureus*, as recently reviewed by Mateo and Jiménez [6].

Most AgNPs showed better antibacterial activity against Gram-negative than against Gram-positive bacteria. This fact is relevant since the shortage of classic antibiotics due to antibiotic resistance caused the World Health Organization (WHO) to assign the highest priority to the search for antibacterial agents against Gram-negative bacteria [94,95]. Moreover, one of the most AgNP-susceptible bacteria was *P. aeruginosa*, with three strains showing similar susceptibility. The same was also observed in our previous work on AgNPs obtained with *Pseudomonas alloputida* culture broths [63] and in other studies, such as when AgNPs produced with a plant extract were tested [96]. In a recent review, the preferred activity of AgNPs against Gram-negative bacteria was sustained [11]. However, this may depend on the AgNPs’ nature and the tested bacterial species or strains, since some studies have also shown the contrary [97,98,99].

Most AgNPs showed MICs indicative of great antibacterial activity, although differences appeared depending on the broths used for their synthesis. The comparison of the antibacterial activity parameters of these AgNPs with others reported in the literature is onerous, since these studies have used various methods and bacterial species or strains for their evaluation. However, we can compare them with those described in our previous work, in which the same bacterial strains and conditions were used for the AgNP activity evaluation [63]. The MIC values obtained for the AgNPs described here were, on average, higher than those previously described, indicating that, in general, these AgNPs are slightly less active, even though, in the case of the E7-AgNPs and L7-AgNPs, they were much less active, and the L4Cl-AgNPs were equally or even more active than some of those described in the previous paper.

Factors such as the Z-potential, shape, and size of the AgNPs affect their antibacterial activity, but correlations among them do not always appear [62,100]. These correlations mainly arise when studying chemically prepared AgNPs with well-established compositions and structures. Thus, from these studies, a negative correlation of the Z-potential sign with activity is usually considered, with AgNPs with negative potentials exhibiting lower activity or small-sized AgNPs showing higher activity. However, for AgNPs synthesized with complex mixtures of biochemical compounds from biological sources, such correlations are not always found. This situation occurred in the current study, with no correlation between the Z-potential or size and activity, although, in our previous report [63], the AgNPs obtained using bacterial broths displayed some size–activity correlation. Neither the hydrodynamic size nor the calculated thickness of the corona correlated with the observed activity levels. Perhaps, for the case of complex nanoparticles, the influences of the several variables that may be involved should be considered together to explain the measured activity levels, which would be a great challenge.

### 3.6. Cytotoxicity

The cytotoxicity of the AgNPs was evaluated on cultured intestinal human cells to estimate the possibility of using them as antibacterial agents, finding different toxicity levels depending on the type of nanoparticles. Cytotoxicity can be evaluated at different levels and using several experimental settings, both in vitro and in vivo. We used well-established tests to obtain complementary information about changes in three relevant cellular endpoints: plasma membrane integrity, metabolic impairment, and lysosomal integrity [101,102]. These tests are frequently used together, in combinations of two or three, to evaluate cytotoxicity in different fields [103,104,105], and their results for our AgNPs in the three tests were generally in agreement.

We did not find a complete correlation between the antibacterial activity of the AgNPs, as represented by their MICs, and their level of cytotoxicity, measured as their IC_30_ on the HCT116 cell line. For example, L4Cl-AgNPs were the most active antibacterial nanoparticles against any tested bacteria and the most cytotoxic. However, because of the low concentration needed to reach the MICs, they appeared non-toxic at this concentration in all of the three cytotoxicity tests performed. On the other hand, E7-AgNPs, with the highest MIC, show relevant cytotoxicity when considering membrane integrity and at the other two endpoints in the case of the MICs for *S. aureus* and *B. subtilis*. Other AgNPs, such as E4-AgNPs and L4-AgNPs, with low but not the lowest toxicity, showed almost no toxicity to the human intestinal cells at the MICs for all the bacteria. Finally, E7Cl-AgNPs, not the most toxic nor the best antibacterial agents, presented lower cytotoxic IC_30_ values than the MICs for *S. aureus* and *B. subtilis*, although, at these MICs, they are highly cytotoxic for human cells.

Many studies do not evaluate the cytotoxicity of the AgNPs, but different toxicity levels have been reported, from very high [23] to no or low cytotoxicity [21,22], when assessed. Thus, the prediction of AgNPs’ cytotoxicity from studies of other AgNPs is not possible, being necessary to measure it for each case. Overall, with our cytotoxicity studies at the AgNPs’ bacterial MICs’, we determined that most of the nanoparticles could work at such concentrations without high toxicity levels. Even with these results, it is necessary to consider that all the assays performed for antibacterial and cytotoxicity assessment were in vitro studies, and their potential extrapolation to in vivo systems is not straightforward.

If considering the possible safety of the use of the AgNPs in terms of the relationship between their toxicity, in terms of their IC_30_s, and their antibacterial activity, in terms of their MICs, the safest AgNPs would be L4-AgNPs for treatment against *E. coli*, *P. aeruginosa*, and *S. epidermidis*; E4-AgNPs against *B. subtilis*; and E4Cl against *K. pneumoniae* and *S. aureus*.

### 3.7. Synergy

One of the most helpful ways to exploit the antimicrobial activity of AgNPs is in combination with classic antibiotics [106]. These combinations may generate a stronger antimicrobial effect than their individual ones, which is called a synergy, and thus allow the reduction of the necessary concentrations of both products used individually to achieve the same antimicrobial activity, which, in some cases, would be able to reduce cytotoxicity [107]. The synergy strategy may also help to recover some antibiotics not in use because of their toxicity or because they have become ineffective due to pathogens’ resistance to them [49,50,51,52,53,54,107,108]. There are several different types of protocols to determine synergy, but we chose the checkerboard assay [67,68] because it produces quantitative results to use comparatively through the calculation of the FICI [68] and MF [49].

Several factors influence the results of synergy studies, some of which are the method and test bacteria used, the antibiotics, and the characteristics of the nanoparticles. In previous studies reviewed by Ribeiro et al. [109], *E. coli* and *S. aureus* were the most frequently tested bacteria, so we used these species for our assays. An exhaustive comparison of our results with the data reported in the literature is difficult because of the use, in different reports, of various experimental methods and strains. In particular, studies based on the disk diffusion method, which is intrinsically a non-quantitative method, are not considered appropriate by many authors [99] since the results may be affected by many non-controllable factors and effective concentrations cannot be determined. The results obtained for our AgNPs using the checkerboard method showed the synergic activity of our AgNPs in combination with Ap against the *E. coli* used by us, but not for the strain of *S. aureus*, and with Tc against *S. aureus*, but not *E. coli*. Km and Sm against both tested bacteria exhibited the highest level of synergy, but the combinations with Cp, Ep, and Nx did not show any positive result. Moreover, in a recent report by our group using the same approach and strains as in the current one, the three antibiotics shared in both studies displayed very similar results, with only slight FICI differences depending on the AgNPs used [63].

The synergy against *P. aeruginosa* of some AgNPs with Ap, Cp, Sm, Tc, and some other antibiotics was studied by Markowska et al. [110], describing positive results for Ap, Sm, and Tc but not for Cp or meropenem (Mp), an antibiotic of the same family as the Ep used by us, also with negative results in our study. Another report has described the synergic behavior of some AgNPs in combination with Ap against an Ap-resistant (Ap^R^) strain of *E. coli* and with gentamicin (Gm), an aminoglycoside antibiotic, against *P. aeruginosa* as well, with similar results, besides additional synergy with Mp. In that report, Tc and Cp also showed a synergy against *S. aureus* [99]. The results of this study are in agreement with our data for the case of the aminoglycoside antibiotic tested by them, Gm, or Sm and Km in this report, but disagree with our positive results of the synergy against *E. coli* when using Tc and the negative one against the Ap^R^ strain of *E. coli* used in the Panáček et al. study. Our evaluation of the AgNPs’ synergy with Tc against *S. aureus* yielded a negative result, with FICI values indicating only an additive effect, but Panáček et al. reported a positive synergy against *S. aureus*. In addition, our AgNPs did not show a synergy with Ap against *E. coli*, but Panáček et al. found one against an ApR strain of this bacterium. Finally, Cp and a carbapenem antibiotic, Mp in their case and Ep in ours, were considered synergic with the AgNPs obtained by Panáček et al. against *S. aureus* and *P. aeruginosa*, respectively, but not with any of our AgNPs against the two bacteria tested. These differences are likely due to the bacterial strains used in each case or to differences in the AgNP characteristics. Other studies have claimed the synergy of Tc with some AgNPs against *S. aureus* [111,112]. However, these studies evaluated the increase in the activity of the combination by the disk diffusion method, a method that does not allow proper quantification based on concentrations.

Finally, the high level of synergy of our AgNPs with Sm or Km against the tested Gram-positive and Gram-negative bacteria is remarkable with respect to those of the rest of the tested antibiotics that showed synergy. Both antibiotics are aminoglycosides, sharing some molecular structures, which may be involved in the observed synergy. Some authors [52,109,113,114] have described significant efficiency in inhibiting bacterial growth of combinations of aminoglycosides and AgNPs. A recent article also reported the synergy of AgNPs with Sm against two strains of *E. coli*, one of *K. pneumoniae*, and another of *P. aeruginosa*, and for Km against *S. aureus* [115]. A synergy with Ap emerged only against *S. aureus* and *P. aeruginosa*. These results show some discrepancies with our results, since our AgNPs behaved synergistically with Sm and Km against *E. coli* and *S. aureus*; they agree with the synergy with Ap against *S. aureus* but not that against *E. coli* found in our work. In another study [53], AgNPs exhibited a synergy with Km against *E. coli* and *S. aureus*, among other species, but no synergy of Ap against any of these species. In the report of Wypij et al. [116], the synergy of AgNPs with Ap, Km, and Tc was assessed, finding positive results for the three antibiotics against *E. coli* and *S. aureus*. Other AgNPs were also synergic with Sm against a Sm-resistant *S. aureus* strain [108].

A recent study using commercial AgNPs and several antibiotics with different modes of action [106] observed no synergy against *E. coli* with ampicillin, targeting cell walls; however, for colistin, also targeting cell envelopes, a 6.9 decrease in the MIC was noted. Moreover, combinations of AgNPs with antibiotics inhibiting protein synthesis, such as the aminoglycoside antibiotics tobramycin, gentamicin, and amikacin, produced a relevant MIC decrease. AgNPs in combination with tetracycline, another antibiotic targeting protein synthesis, did not show a significant MIC decrease. Our results with aminoglycosides and Ap against *E. coli* agree with the results of this report, but discrepancies exist for tetracycline. Panáček et al. [99] studied the synergy of AgNPs with many antibiotics of different structural classes, and most of them showed a relevant decrease in antibiotics’ MICs at AgNP concentrations below their MICs. The results suggested an unspecific synergy between the AgNPs and the antibiotics. The available data indicate that a correlation between the antibiotic target and the observed synergy does not exist, even though the mechanism of each antibiotic targeting a particular process may be proprietary, so it would be necessary to carry out more precise studies to clarify this matter. The studies on the synergy of AgNPs with antibiotics against other ESKAPE species, such as *Acinetobacter baumannii*, *P. aeruginosa*, and *S. aureus*, reviewed by Mateo and Jiménez [6], show similar results, with some agreements and discrepancies with our results, which may depend on the AgNPs’ characteristics, the method of analysis, and the strains or species used for testing. Using different AgNPs and bacterial test strains may affect the results of synergy studies. Because of this, an agreement on standards would be advisable, at least in the procedures and the bacterial strains used, to make the results comparable.

Although all our AgNPs showed a similar result in a general view, regarding positive or negative synergy, the FICI values showed some differences depending on the AgNP type. In our previous work using the same conditions for the assays but AgNPs produced with bacterial broths [63], the same results were obtained for the antibiotics tested in both studies, with the range of FICI values quite similar for Ap as well as for Nx, for both groups of AgNPs. However, in general, they showed lower values, suggesting a higher level of synergy, for the AgNPs obtained using bacterial broths: 0.039–0.180 vs. 0.047–0.313. When using different types of biogenic AgNPs, the levels of synergy can be different depending on the method and the conditions of synthesis, even when using biological material from the same microorganism for AgNP synthesis, and also depending on the type of microorganism itself, mainly in the cases of high levels of synergy.

Nowadays, the mechanisms involved in the synergy of AgNPs with antibiotics are unknown, but some studies state that, in some cases, they may be due to membrane permeability changes produced by AgNPs, facilitating the entrance of the antibiotic [53]. Some authors have studied, by FTIR, the possibility of covalent bonding between the AgNPs and the antibiotics, with negative results, but they detected, by TEM, the agglomeration of the AgNPs in the presence of Km or Ap, as well as an increase in the AgNPs’ size by DLS analysis. Since they did not observe a synergy for the AgNP–Ap combination, the observed agglomeration did not seem to be involved in the synergy mechanism, at least with this antibiotic. Another study also suggested the formation of AgNP–antibiotic complexes for some β-lactam antibiotics [117,118].

In a report, Deng et al. [119] suggested the formation of AgNP–antibiotic complexes that could interact with the bacterial envelopes for the more effective release of Ag^+^ from the AgNP in the cases of Km, neomycin (Nm), and Tc, based on the observation of changes in the UV–Vis and Raman spectra of the AgNPs in combination with the antibiotics. In addition, Vazquez-Muñoz et al. reported [53] the agglomeration of a particular type of AgNP in the presence of Km or Ap. The solvent used was water in both studies, and the concentrations of the two components were higher than those producing detectable synergic activity. Since the interactions would depend, besides the characteristics of the two components, on their concentrations and the medium in which they interact, the results shown by these reports, which were well sustained, may not be representative of what occurs in synergy experiments. Even if these interactions occurred in the tested conditions, the mechanism by which they can affect the activity of the combinations remains unknown and would require further study for clarification.

To obtain insights into the mechanism of synergy, we tested the effect of the solvent and levels of concentrations in the AgNP–antibiotic interactions by employing one of the techniques used by Deng et al. [119], namely UV–Vis spectroscopy, and another used by Vazquez-Muñoz et al. [53], namely DLS, to further study the agglomeration of AgNPs in the presence of antibiotics. We found a reduction in the peak at the λ_max_ of the AgNPs incubated with the tested antibiotics when using a sufficiently high concentration of the AgNPs to obtain a spectrum with the necessary absorbance level to allow UV–Vis detection and a high concentration of the antibiotic. The UV–Vis spectra of the mixes prepared in water showed AgNP aggregation and a decrease in the absorbance at λ_max_ but without aggregation for those produced in the culture medium. These results would indicate that, in the presence of the culture medium, AgNP aggregation, even in the high concentrations used, did not take place, while the decrease in absorbance occurring for all the tested AgNPs with all the tested antibiotics may have been due to some quenching effect of the medium. Analyzing the results of DLS, the mixes produced in water at high concentrations of AgNPs and antibiotics displayed a size increase in the nanoparticles in the case of the antibiotics producing the maximum synergy. However, for the mixes prepared in the nutritive medium, the AgNPs only showed a slight size increase, related to synergy only in the case of Sm. It is impossible to perform UV–Vis analysis for the concentration conditions in which synergy occurs, because the low concentrations of AgNPs do not allow the required absorbance level. Nevertheless, the DLS analyses performed in these conditions evidenced no significant size change in the AgNPs, even in water, except for Sm, but not for Km. Some AgNPs showed small size increments with certain antibiotics. These data suggest that the results could change depending on the analysis conditions, the water or medium used, and the concentrations used. In the conditions in which synergy is detected for some and not for other antibiotics, the differential formation of aggregated AgNPs is not supported as the cause of the observed synergy.

### 3.8. ROS and Bacterial Killing by the AgNPs

ROS production is one of the factors that seems to be involved in the antibacterial activity of silver nanoparticles [120,121], while its relationship with bacterial killing is not yet clearly established. The importance of this phenomenon is controversial, considered as the cause of bacterial death by AgNPs or other forms of silver, or with other effects [122]. We wished to test whether the production of ROS paralleled the decrease in viable cells in bacterial cultures challenged with some of the AgNPs reported here. The results obtained after a 4 h treatment showed an increase in ROS accumulation in *E. coli* with certain levels of the tested AgNP concentrations, but with small variations in the accumulated ROS in the case of *S. aureus*. This effect could be due to differences in the methods of ROS detoxification that *S. aureus* or *E. coli* might implement. Our results may agree with those from a study in which a weak effect on ROS production by different strains of *S. aureus* appeared when treated with linezolid, an antibiotic producer of large amounts of ROS in other species [123]. The assessment of the viable cells present in the bacterial samples after incubation with the AgNPs showed a decrease depending on the AgNP concentration. The maximum ROS accumulation appeared at AgNP concentrations in which none or a low level of viable cells remained. The patterns of viable cells decreasing and the ROS accumulation increasing were different depending on the presence of chloride in the broths used for AgNP synthesis. For L4Cl-AgNPs and L7Cl AgNPs at low concentrations, the decrease in viable cells did not correlate with the low level of ROS increase. Thus, ROS production does not seem to be the main factor affecting the killing of the bacteria by these AgNPs. In other reports measuring the ROS production and bacterial toxicity of other metal nanomaterials, a lack of correlation between ROS production and bacterial death was evident, indicating the existence of other factors involved, such as interaction with the bacterial membranes [124]. For AgNPs prepared with broths without chloride, the decrease in viable cells mostly followed the increase in ROS production, suggesting a relevant effect of ROS in the rate of bacterial killing by these AgNPs. The fact that the MICs of the AgNPs against *S. aureus* were higher than for *E. coli* could agree with the lower production of ROS observed by the former. In some studies, differential effects on macromolecules’ oxidation promoted by ROS after treatment with other metal nanomaterials were observed depending on the bacteria, *E. coli* or *S. aureus*, perhaps indicating different strategies by which these two bacteria cope with ROS accumulation [125]. In addition, in a study, the lower activity of AgNPs against *S. aureus* was considered to be due to structural differences in the cellular envelopes [126], but further studies including other possible factors influencing the killing of bacteria, such as membrane permeability changes or DNA damage, are needed to fully elucidate the mechanism of action of AgNPs.

## 4. Materials and Methods

### 4.1. Microorganisms and Culture Media Used

The microalga used for this study was an isolate from the Tinto River estuary (Huelva, Spain). Media used to culture the microalga were BG-11 [127] and BG-11A (BG-11 with the replacement of ${CaCl}_{2}\cdot {2H}_{2}O$ and ${MnCl}_{2}\cdot {4H}_{2}O$ with ${Ca(NO}_{3}{)}_{2}$ and ${MnSO}_{4}$, respectively, maintaining the molar concentrations of the cations and using $H_{2}{SO}_{4}$ to set the pH to 4 or 7).

To evaluate the AgNPs’ antibacterial activity, we used six species: *Bacillus subtilis* 168, *Staphylococcus aureus* CECT 794 and *Staphylococcus epidermidis* ATCC 12228, as Gram-positive bacteria, and *Escherichia coli* ATCC 25922, *Klebsiella pneumoniae* ATCC 29665, and *Pseudomonas aeruginosa* CECT 108, PA01, and PA14, as Gram-negative ones. Bacteria grew in nutritive medium (3 g/L meat extract (Merck Millipore, Darmstadt, Germany), 5 g/L bacteriological peptone (Condalab, Torrejón de Ardoz, Spain), 5 g/L NaCl (Merck Millipore) (with 15 g/L of European bacteriological agar [Condalab] for nutritive agar)) at 37 °C in a Gyrotory^®^ Water Bath Shaker model G76 (New Brunswick Scientific Co., Inc., Edison, NJ, USA) with shaking at 150 rpm.

The microalga was grown in cultures performed at 20 °C in a 16/8 light/dark cycle, using five Osram-Dulux 11 W fluorescent light tubes (Milano, Italy), by quadruplicate in 250 mL Erlenmeyer flasks containing 120 mL of BG-11 or BG-11A at pH 4 or 7. Growth was followed spectroscopically (300–700 nm) on samples measured in a FLUOStar^®^ Omega (BMG, Labtech, Offenburg, Germany) in microtiter 96-well plates using 200 µL of the culture. We collected samples in the early (OD680 ≈ 1) and late (OD680 ≈ 2) growth phases for each medium and pH.

### 4.2. Cell-Free Broth Preparation

To obtain the cell-free broths from the alga cultures, they were centrifugated for 30 min at 4 °C in a Beckman Coulter Allegra X-12R Centrifuge (Fullerton, CA, USA) at 2105× *g*. Afterwards, the supernatants were filtered through 0.22 µm pore size Millex^®^ filters (Merck-Millipore, Cork, Ireland) to obtain the cell-free material. Broths prepared from the quadruplicated cultures were mixed and kept at −20 °C until use.

### 4.3. Green Synthesis of AgNPs

For the synthesis of AgNPs, we used the same techniques and protocols described before [63]. Briefly, the synthesis reactions were set by adding 20 mM ${AgNO}_{3}$ (Merck) to the cell-free broths to a final 1 mM silver concentration and irradiating them (three Sylvania 18 W tubes 15 cm above the sample) at 22 °C until reaching the maximum absorbance.

Afterwards, the AgNPs were sedimented, washed with MilliQ water, sedimented again, and finally resuspended in MilliQ water and kept at 4 °C for no more than one week until used.

Acronyms for the different types of nanoparticles were, depending on the growth phase (early (E) and late (L) phases), the pH (4 or 7), and the medium (BG-11 (with Cl), BG-11A (without Cl), E4Cl-AgNPs, L4Cl-AgNPs, E4-AgNPs, L4-AgNPs, E7Cl-AgNPs, L7Cl-AgNPs, E7-AgNPs, and L7-AgNPs.

To analyze the AgNPs produced at different reaction times, L4Cl-AgNPs and L4-AgNPs were sampled at several time points during their synthesis, and the AgNPs were washed in the usual way [63] before their characterization.

To evaluate the possibility of producing new batches of AgNPs with broths previously used for their synthesis, L4Cl-AgNPs and L7Cl-AgNPs were recovered by centrifugation from the first synthesis in the usual conditions and the supernatants used to set up the following round of synthesis in the same conditions as in the first one, collecting and washing the AgNPs in the same way in all the reaction rounds.

### 4.4. Characterization of the AgNPs

#### 4.4.1. Determination of the AgNPs’ Physicochemical Properties

We performed the physicochemical characterization of the silver nanoparticles as previously described [63]. UV–Vis spectra were registered using a FLUOStar^®^ Omega (BMG, Labtech, Offenburg, Germany). The size and shape of AgNPs were determined by transmission electron microscopy (TEM) with a JEM1400 JEOL microscope (Tokyo, Japan). For the nanoparticle diameter assessment, at least 1000 particles of each AgNP type from TEM images were analyzed using the ImageJ64 program (https://imagej.nih.gov/ij/download.html (accessed on 1 February 2023)). The zeta potential and the hydrodynamic diameter of AgNPs were measured by dynamic light scattering (DLS) using a Zetasizer Ultra (Malvern Panalytical, Malvern, UK). Fourier transform infrared spectroscopy (FTIR) analyses were carried out in the range of 4000–500 cm^−1^ using a Spectrum Two (Perkin-Elmer, Waltham, MA, USA) for macro-analysis and a Spotlight 200 (Perkin-Elmer) for micro-analysis. The crystallinity of the AgNPs was assessed by X-ray diffraction (XRD) with an X’Pert PRO theta/2theta X-ray diffractometer (Malvern Panalytical, Malvern, UK), with a primary germanium monochromator (monochromator Johansson) and an X’Celerator fast detector. The silver concentrations of nanoparticle suspensions and their compositions were determined using an S2 PicoFox TXRF spectrometer (Bruker, Ettlingen, Germany) with a Mo X-ray source, an XFlash SDD detector, and a multilayer monochromator. Throughout this paper, the concentration of AgNPs is expressed as the silver concentration.

#### 4.4.2. Evaluation of the AgNPs’ Antibacterial Activity

We used the quantitative microdilution method for the AgNPs’ antibacterial activity determination. Cultures with and without AgNPs (as a negative control) were set in triplicate in 96-well microplates (Sarsted, Nümbrecht, Germany) and incubated overnight at 37 °C, while hourly registering the absorbance at 660 nm. We used the violet crystal assay for the antibiofilm activity assessment. The antibacterial activity evaluation was as in a previous report [63]. IC_50_ and ICb_50_ are expressed as the average value ± the standard deviation.

#### 4.4.3. Assessment of the AgNPs’ Cytotoxicity on Cultured Human Cells

Human cell line HTC116 (ATCC CCL-247), derived from colorectal carcinoma, was used to study the cytotoxicity of the AgNPs. Cells were maintained at 37 °C under a humified atmosphere with 5% ${CO}_{2}$ in McCoy´s 5a medium modified with stable L-glutamine (HyClone™, Cytiva Europe GmbH, Freiburg im Breisgau, Germany) and supplemented with 10% fetal bovine serum (FBS; Gibco, Thermo Fisher Scientific, Waltham, MA, USA) and 1% penicillin–streptomycin (HyClone™, Cytiva). The cultures were set on ventilated F75 vessels (Fisher Scientific Spain, Madrid, Spain), renewing the medium twice a week and subculturing the cells before reaching 100% confluence, using 0.25% trypsin and phosphate-buffered saline (PBS; HyClone™ Cytiva) for cell detachment. Daily control of the cell cultures was conducted using a phase-contrast microscope (Leica DMi1, Leica Microsystems, Wetzlar, Germany).

HCT116 cells were seeded in 96-well plates at 1.5 × 10^5^ cells/mL density and grown overnight before exposure for 24 h to increasing concentrations of the AgNPs in the medium with 10% FBS. The AgNPs’ concentration ranges were selected ad hoc based on the bacterial MIC values previously obtained and were as follows: E4Cl-AgNPs (0.4–25.6 µg/mL), E7Cl-AgNPs (0.4–6.4 µg/mL), L4Cl-AgNPs (0.4–10 µg/mL), L7Cl-AgNPs (0.4–25.6 µg/mL), E4-AgNPs (0.5–25 µg/mL), E7-AgNPs (1–25 µg/mL), L4-AgNPs (0.5–25 µg/mL), L7-AgNPs (0.4–40 µg/mL). The cytotoxicity of ${AgNO}_{3}$ was assessed under the same circumstances for comparative purposes.

Cells of different passage numbers, always being below 20, were used in three independent experiments. At experiment termination, cell cultures were assayed for metabolic activity (Alamar blue, AB), plasma membrane integrity (carboxyfluorescein diacetate acetoxymethyl ester, CFDA-AM), and lysosome integrity (neutral red, NR), following the method of Schirmer et al. (1997) [102] with slight modifications. Briefly, after a washing step in PBS, cells were incubated for 30 min with a combination of 5% AB *v*/*v* (Invitrogen, ThermoFisher Scientific, Waltham, MA, USA) and 4 µM CFDA-AM (Invitrogen) in DMEM without phenol red (HyClone™ Cytiva). Fluorescence was measured at the appropriate excitation/emission wavelengths (530/595 nm for AB, 485/530 nm for CFDA-AM) using a plate reader (Tecan Infinite 200 PRO, TECAN, Zürich, Switzerland). Next, after discarding the AB/CFDA-AM solution, cells were further incubated for 1 h with 40 µg/mL of NR in DMEM without phenol red. Afterwards, cells were fixed (1% *w*/*v* (Sigma-Aldrich Co., St. Louis, MO, USA) and 0.37% *v*/*v* formaldehyde (Panreac Química S.L.U., Barcelona, Spain) in $H_{2}O$) and NR was extracted (1% acetic acid and 50% ethanol (both from Panreac Química S.L.U.)) with gentle shaking for 10 min. Fluorescence was measured at 530/640 nm excitation/emission wavelengths using the same plate reader. The parameter IC_30_, corresponding to 70% of viable cells, was used for cytotoxicity comparisons, as recommended by the ISO 10993-5:2009(E) standard [128].

#### 4.4.4. Assessment of the Synergy between AgNPs and Classic Antibiotics

For synergy evaluation, we used the checkerboard assay [67,68]. Ampicillin and streptomycin (Duchefa Biochemie, Haarlem, The Netherlands) together with ciprofloxacin, kanamycin, nalidixic acid, and tetracycline (Sigma-Aldrich Co., St. Louis, MO, USA) and ertapenem (Merck KGaA, Darmstadt, Germany) were the antibiotics tested. The bacteria used in these analyses were *E. coli* ATCC 25922 and *S. aureus* CECT 794. The experimental methodology was as previously described [63], and the assay was carried out in duplicate, incubating cultures in 96-well microplates for 24 h at 37 °C. For synergy level evaluation, we used the fractional inhibitory concentration index (FICI) [67,68] and the modulatory factor (MF) [49].

##### Analysis of the Interaction of AgNPs and Antibiotics

The mixes analyzed to assess the effects of the antibiotics on the AgNPs’ spectra contained AgNPs at the concentrations that allowed their UV–Vis detection (final concentrations were 7.46, 14.75, 26.75, and 10.52 µg/mL for L4Cl-AgNPs, L7Cl-AgNPs, L4-AgNPs, and L7-AgNPs, respectively) and each antibiotic at 50 µg/mL, in Milli Q water or nutritive medium. We used the same microtiter plate reader employed in the synergy studies to register the spectra.

In the DLS analysis, we used the high-concentration solutions prepared above and others at the synergy concentrations in which the AgNPs’ and antibiotics’ final concentrations were 0.47, 0.46, 1.84, and 1.32 µg/mL for L4Cl-AgNPs, L7Cl-AgNPs, L4-AgNPs, and L7-AgNPs, respectively; for the antibiotics, the concentrations were Ap 4.00 µg/mL, Cp 0.50 µg/mL, Ep 0.13 µg/mL, Km 0.40 µg/mL, Sm 0.50 µg/mL, and Tc 0.25 µg/mL. DLS measurements were carried out in triplicate after 3 min sonication of the solutions, using the same DLS equipment mentioned above.

### 4.5. Reactive Oxygen Species (ROS) Production by the AgNPs and Their Bacterial Killing Rate

ROS production by *E. coli* ATCC 25922 and *S. aureus* CECT 794, in the presence of several concentrations of each AgNP, was determined by the rise in fluorescence during the incubation time from 2′-7′-dichlorodihydrofluorescein diacetate (DCFH-DA) (Sigma-Aldrich, Co., St. Louis, MO, USA). We performed these experiments as previously described [63]. To evaluate the bacteria-killing capability of the AgNPs and to correlate it with ROS production, we quantified the viable cells in the cultures after the treatment with AgNPs by plating 25 µL of a 1/20,000 dilution of the culture in nutritive agar and counting colonies after o/n incubation at 37 °C.

### 4.6. Statistical Analysis

The software used to visualize the results and for statistical analyses was GraphPad Prism 8 (GraphPad Software, San Diego, CA, USA), assessing the differences between the IC_50_ and ICb_50_ of each AgNP with a two-tailed unpaired *t*-test and a 0.05 *p*-value, attending to the variables of the broth used for AgNP synthesis, the growth phases of the cultures, the presence or not of Cl^−^, and the pH of the media used to set the cultures (Table 2).

We used a one-way ANOVA with Dunnett´s multiple comparisons and a 0.05 *p*-value for comparisons of the antibacterial activity of the AgNPs obtained at different synthesis times (Table 3) and for those of the AgNP batches from successive syntheses with the same broth (Table 4). The same procedure was employed to determine the significant differences from the control in terms of the hydrodynamic diameters of the AgNPs in combination with antibiotics (Figure 11).

A two-way ANOVA and Dunnett’s multiple comparisons, with a *p*-value of 0.05, were used to determine the significance of the differences in the ROS accumulated after 4 h of treatment of the bacteria with the AgNPs and that of the untreated control, as well as between each value and that corresponding to the immediate lower concentration of AgNPs. We used the same method to evaluate the significance of the bacterial viability differences depending on the AgNP concentrations (Figure 12).

For cytotoxicity assays, all the statistical analyses, including normality and homoscedasticity evaluation, appropriate tests for ANOVA or Kruskal–Wallis and multiple comparisons, and sigmoidal dose–response curves with variable slope to the estimate effective concentration (Figure S3), as well as IC_30_ determination (Table 5), were carried out using the same software.

## 5. Conclusions

In this work, we obtained eight types of silver nanoparticles using broths from cultures in different conditions of a microalga. The synthesis of each AgNP type followed different kinetics, and the early synthesis stages produced less stable and more active AgNPs but with lower yields. The broths used to obtain the first AgNP batch could generate a second one with similar efficiency, synthesis kinetics, and antibacterial activity.

The nanoparticles obtained at reaction times producing the maximum yields were quasi-spherical, with small core diameters ranging from 17 to 21 nm. They crystallized in cubic face-centered structures containing Ag^0^ crystals and, in the case of AgNPs synthesized with broths containing Cl^−^, also AgCl ones in lower concentrations. The AgNPs’ hydrodynamic diameters and Z-potentials were 63.9 to 128.5 nm and −22.4 to −36.9 mV, respectively. The nanoparticles’ coronas showed subtle differences in composition as determined by FTIR, with different proportions of proteins, carbohydrates, and aliphatic substances.

Most AgNPs showed very high antibacterial activity, with variations in the activity parameters depending on the type of AgNPs; in general, their cytotoxicity to human cultured cells was low or absent at the AgNP concentrations corresponding to the MICs against the tested bacteria. No correlation appeared between the physicochemical properties of the AgNPs and their antibacterial activity. The AgNPs with the best antibacterial activity against any of the tested bacteria were those prepared with broths from late-phase cultures initiated at pH 4 in a medium containing Cl^−^ (L4Cl-AgNPs).

All AgNPs could act synergistically with Sm or Km against *E. coli* and *S. aureus*, with some differences in the FICI values depending on the AgNP type. Moreover, a synergy was observed with Tc against *E. coli* and Ap against *S. aureus*. The physicochemical properties of the AgNPs and their MICs or MBCs did not correlate with the synergy levels observed, but the L7Cl-AgNPs, prepared from late-phase cultures initiated at pH 7 in a medium with Cl^−^, showed the highest levels of synergy with the four antibiotics producing any synergy (Ap, Km, Sm, and Tc).

The AgNPs obtained in this work produced ROS accumulation in *E. coli* and less in *S. aureus*. For some AgNPs, the ROS accumulation correlated with the viability decrease of these bacteria, indicating a relevant role of ROS in the bacteria killing by these AgNPs. However, such a correlation was not apparent for other AgNPs, thus indicating that other mechanisms still to be studied could also contribute to their bacteria-killing activity.

It has been shown that the AgNPs obtained in this work could help in addressing the antibiotic resistance problem in some uses alone or in synergic combinations with antibiotics, and some aspects of the mechanisms of synergy with antibiotics and the relationship between the ROS production and the bacterial killing by the AgNPs have been clarified.

## Figures and Tables

**Table 1:** Physicochemical parameters of the AgNPs.

Nanoparticles (AgNPs)	Z-Potential (mV)	Diameter (DLS) (nm)	PDI (DLS)	Diameter (TEM) (nm)	PDI (TEM)
**E4Cl-AgNPs**	−36.88 ± 1.03	94.44 ± 2.74	0.29	14.88 ± 8.02	0.29
**L4Cl-AgNPs**	−26.12 ± 0.81	75.58 ± 1.66	0.47	12.19 ± 5.93	0.24
**E4-AgNPs**	−24.71 ± 2.19	98.53 ± 5.79	0.48	11.01 ± 6.45	0.34
**L4-AgNPs**	−22.41 ± 2.66	83.20 ± 2.06	0.30	7.02 ± 3.19	0.21
**E7Cl-AgNPs**	−35.37 ± 1.17	128.50 ± 3.98	0.42	20.41 ± 15.04	0.54
**L7Cl-AgNPs**	−27.25 ± 1.28	63.88 ± 0.86	0.37	14.28 ± 10.87	0.58
**E7-AgNPs**	−31.86 ± 0.16	79.20 ± 0.55	0.37	10.95 ± 7.03	0.41
**L7-AgNPs**	−29.86 ± 0.11	89.16 ± 5.39	0.30	10.65 ± 6.07	0.32

**Table 2:** Parameters for antibacterial, antibiofilm, and cytotoxic activity of the AgNPs.

Bacteria	AgNPs	MIC (μg/mL)	MBC (μg/mL)	IC_50_ (μg/mL)	ICb_50_ (μg/mL)	MIC_AB (%)	MIC_CFDA-AM (%)	MIC_NRU (%)
***E. coli* ATCC 25922**	**Sm**	16.00	16.00	5.40 ± 1.06	5.83 ± 1.52	-	-	-
	**AgNO_3_**	0.53	0.53	0.15 ± 0.01	0.33 ± 0.03	100.00 ± 0.00	93.39 ± 0.00	100.00 ± 0.00
	**E4Cl-AgNPs**	1.25	1.25	0.31 ± 0.07 */#	0.56 ± 0.07 */#/•	93.63 ± 2.58	89.32 ± 3.99	99.77 ± 0.69
	**L4Cl-AgNPs**	0.47	0.47	0.14 ± 0.05 */#/•	0.29 ± 0.05 */#/•	97.56 ± 2.13	94.90 ±2.79	99.94 ± 0.07
	**E4-AgNPs**	2.64	2.64	1.04 ± 0.17 #	1.75 ± 0.10 #/•	96.67 ± 3.32	87.69 ± 4.45	100.00 ± 0.00
	**L4-AgNPs**	1.84	1.84	0.99 ± 0.03 #	1.47 ± 0.41 #	95.56 ± 2.65	93.38 ± 4.17	100.00 ±0.00
	**E7Cl-AgNPs**	1.73	1.73	0.40 ± 0.09 #	0.79 ± 0.11 */#/•	76.39 ± 4.89	87.54 ± 6.00	89.52 ± 5.05
	**L7Cl-AgNPs**	0.84	0.84	0.43 ± 0.16 •	0.43 ± 0.01 */#/•	87.77 ± 4.45	89.92 ± 5.29	99.94 ± 0.20
	**E7-AgNPs**	5.78	5.78	1.44 ± 0.27 #	3.83 ± 0.34 */#/•	87.53 ± 4.12	68.76 ± 5.09	99.82 ± 1.04
	**L7-AgNPs**	2.63	2.63	0.87 ± 0.28	1.91 ± 0.45 */#	99.99 ± 0.03	97.90 ± 1.90	100.00 ± 0.01
***K. pneumoniae* ATCC 29665**	**Sm**	4.00	4.00	2.59 ± 0.18	2.29 ± 0.38	-	-	-
	**AgNO_3_**	0.53	0.53	0.27 ± 0.01	0.30 ± 0.07	100.00 ± 0.00	93.39 ± 0.00	100.00 ± 0.00
	**E4Cl-AgNPs**	1.25	1.25	0.51 ± 0.16 #	0.76 ± 0.13 #	93.63 ± 2.58	89.32 ± 3.99	99.77 ± 0.69
	**L4Cl-AgNPs**	0.93	0.93	0.46 ± 0.10 #/•	0.66 ± 0.10 #/•	89.03 ± 5.28	86.74 ± 4.65	98.88 ± 0.71
	**E4-AgNPs**	5.27	5.27	1.49 ± 0.12 */#/•	2.24 ± 0.74 */#/•	95.46 ± 3.07	84.83 ± 4.27	100.00 ± 0.00
	**L4-AgNPs**	3.69	3.69	0.93 ± 0.12 */#/•	0.98 ± 0.25 */#/•	94.22 ± 2.47	90.30 ± 4.09	100.00 ± 0.00
	**E7Cl-AgNPs**	1.73	1.73	0.92 ± 0.48 #	0.90 ± 0.10 #	76.39 ± 4.89	87.54 ± 6.00	89.52 ± 5.05
	**L7Cl-AgNPs**	1.67	1.67	1.00 ± 0.19 #/•	0.99 ± 0.03 #/•	77.39 ± 5.08	82.28 ± 6.17	99.49 ± 1.13
	**E7-AgNPs**	11.56	23.13	6.93 ± 0.80 */#/•	8.41 ± 1.32 #/•	76.06 ± 3.97	55.87 ± 4.32	96.78 ± 9.54
	**L7-AgNPs**	10.53	21.05	3.86 ± 0.42 */#/•	4.73 ± 2.02 #/•	96.97 ± 2.88	73.11 ± 6.85	96.28 ± 5.92
***P. aeruginosa* CECT 108**	**Sm**	16.00	16.00	4.50 ± 0.27	6.43 ± 0.75	-	-	-
	**AgNO_3_**	0.27	0.53	0.03 ± 0.01	0.16 ± 0.02	100.00 ± 0.00	93.39 ± 0.00	100.00 ± 0.00
	**E4Cl-AgNPs**	0.63	0.63	0.15 ± 0.03 #/•	0.36 ± 0.07 #/•	96.05 ± 2.10	92.83 ± 3.58	99.93 ± 0.25
	**L4Cl-AgNPs**	0.47	>0.93	0.18 ± 0.03 #/•	0.36 ± 0.07 */•	97.56 ± 2.13	94.90 ± 2.79	99.94 ± 0.07
	**E4-AgNPs**	1.32	2.64	0.08 ± 0.02 */#/•	0.34 ± 0.02 */#/•	97.57 ± 3.36	90.08 ± 4.78	100.00 ± 0.00
	**L4-AgNPs**	0.92	3.69	0.28 ± 0.04 */#/•	0.70 ± 0.12 */#/•	96.60 ± 2.67	95.52 ± 3.81	100.00 ± 0.00
	**E7Cl-AgNPs**	0.87	0.87	0.55 ± 0.23 */#/•	0.56 ± 0.08 */#/•	95.82 ± 2.16	96.48 ± 3.00	98.90 ± 1.09
	**L7Cl-AgNPs**	0.84	0.84	0.07 ± 0.02 */#/•	0.73 ± 0.06 */#/•	87.77 ± 4.45	89.92 ± 5.29	99.94 ± 0.20
	**E7-AgNPs**	11.56	>23.13	1.62 ± 0.77 #/•	6.38 ± 0.54 */#/•	76.06 ± 3.97	55.87 ± 4.32	96.78 ± 9.54
	**L7-AgNPs**	2.63	2.63	0.77 ± 0.17 #/•	1.45 ± 0.04 */#/•	100.00 ± 0.03	97.90 ± 1.90	100.00 ± 0.01
***S. aureus* CECT 794**	**Sm**	32.00	32.00	4.15 ± 0.11	5.45 ± 0.92	-	-	-
	**AgNO_3_**	2.12	4.24	0.56 ± 0.02	1.28 ± 0.15	100.00 ± 0.00	93.39 ± 0.00	100.00 ± 0.00
	**E4Cl-AgNPs**	5.00	5.00	2.68 ± 0.38 */#/•	3.07 ± 0.42 */•	84.13 ± 3.03	77.58 ± 4.16	97.36 ± 4.09
	**L4Cl-AgNPs**	1.87	3.73	0.72 ± 0.05 */#/•	0.98 ± 0.11 */#/•	61.33 ± 5.41	69.19 ± 5.23	81.07 ± 3.19
	**E4-AgNPs**	10.54	10.54	3.74 ± 0.40 */#/•	4.39 ± 2.32 •	93.83 ± 3.26	81.43 ± 4.95	100.00 ± 0.00
	**L4-AgNPs**	14.74	29.48	7.71 ± 0.71 */#/•	7.61 ± 0.25 #/•	90.38 ± 2.78	80.32 ± 3.67	100.00 ± 0.00
	**E7Cl-AgNPs**	3.47	3.47	1.02 ± 0.09 */#/•	1.59 ± 0.12 #/•	30.79 ± 5.92	63.91 ± 6.61	44.08 ± 7.00
	**L7Cl-AgNPs**	3.43	6.69	1.53 ± 0.10 */#/•	2.13 ± 0.35 #/•	61.18 ± 4.99	70.08 ± 6.14	95.35 ± 4.81
	**E7-AgNPs**	23.13	46.25	6.39 ± 0.64 #/•	11.51 ± 1.58 #/•	58.97 ± 4.75	42.13 ± 5.00	61.87 ± 8.28
	**L7-AgNPs**	21.05	21.05	6.03 ± 0.02 #/•	10.15 ± 0.28 #/•	66.41 ± 11.00	39.70 ± 7.81	33.27 ± 23.90
***S. epidermidis* ATCC 12228**	**Sm**	>256.0	>256.0	>256.0	>256.0	-	-	-
	**AgNO_3_**	1.06	4.24	0.27 ± 0.01	0.90 ± 0.35	100.00 ± 0.00	93.39 ± 0.00	100.00 ± 0.00
	**E4Cl-AgNPs**	2.50	10.00	0.49 ± 0.11 #	0.80 ± 0.35	89.82 ± 2.91	84.32 ± 4.12	99.21 ± 1.78
	**L4Cl-AgNPs**	0.93	3.73	0.31 ± 0.08 #	0.55 ± 0.08 #/•	89.03 ± 5.28	86.74 ± 4.65	98.88 ± 0.71
	**E4-AgNPs**	2.64	10.54	1.27 ± 0.37 #/•	1.40 ± 0.20 •	96.67 ± 3.32	87.69 ± 4.45	100.00 ± 0.00
	**L4-AgNPs**	1.84	14.74	1.25 ± 0.23 #	1.61 ± 0.26 #	95.56 ± 2.65	93.38 ± 4.17	100.00 ± 0.00
	**E7Cl-AgNPs**	1.73	3.47	0.42 ± 0.09 #	0.81 ± 0.05 #	76.39 ± 4.89	87.54 ± 6.00	89.52 ± 5.05
	**L7Cl-AgNPs**	1.67	3.34	0.47 ± 0.09 #	0.82 ± 0.10 #/•	77.39 ± 5.08	82.28 ± 6.17	99.49 ± 1.13
	**E7-AgNPs**	11.56	23.13	2.27 ± 0.28 */#/•	5.28 ± 0.19 */#/•	76.06 ± 3.97	55.87 ± 4.32	96.78 ± 9.54
	**L7-AgNPs**	5.26	42.10	1.30 ± 0.33 */#	1.57 ± 0.01 */#	99.81 ± 0.31	91.85 ± 4.55	99.93 ± 0.32
***B. subtilis* 168**	**Sm**	64.00	>256.00	7.58 ± 0.82	14.09 ± 5.80	-	-	-
	**AgNO_3_**	1.06	1.06	0.27 ± 0.08	0.40 ± 0.13	100.00 ± 0.00	93.39 ± 0.00	100.00 ± 0.00
	**E4Cl-AgNPs**	5.00	5.00	1.40 ± 0.31 */#/•	2.01 ± 0.34 */•	84.13 ± 3.03	77.58 ± 4.16	97.36 ± 4.09
	**L4Cl-AgNPs**	1.87	7.46	0.58 ± 0.06 */#/•	0.84 ± 0.15 */#/•	61.33 ± 5.41	69.19 ± 5.23	81.07 ± 3.19
	**E4-AgNPs**	2.64	10.54	0.76 ± 0.18 */#/•	1.26 ± 0.45 */•	96.67 ± 3.32	87.69 ± 4.45	100.00 ± 0.00
	**L4-AgNPs**	7.37	7.37	4.14 ± 1.29 */#	6.37 ± 0.75 */#/•	92.53 ± 2.29	86.05 ± 3.51	100.00 ± 0.00
	**E7Cl-AgNPs**	3.34	3.34	0.45 ± 0.03 */#/•	0.86 ± 0.01 */#/•	33.16 ± 5.91	65.63 ± 6.66	47.33 ± 6.99
	**L7Cl-AgNPs**	3.34	3.34	1.06 ± 0.10 */#/•	1.28 ± 0.18 */#/•	61.86 ± 4.99	70.60 ± 6.16	95.71 ± 4.63
	**E7-AgNPs**	23.13	23.13	4.57 ± 1.71 #/•	6.20 ± 0.35 */#/•	58.97 ± 4.75	42.13 ± 5.00	61.87 ± 8.28
	**L7-AgNPs**	10.52	21.05	3.14 ± 0.49 #	3.35 ± 0.83 */#/•	96.98 ± 2.87	73.15 ± 6.85	96.30 ± 5.91

MIC_AB; MIC_CFDA-AM; and MIC_NRU: % of viable cells at the bacterial MICs according to cytotoxicity curves; (*) significant differences between AgNPs prepared from the medium with the same chlorine concentration but different growth phase; (#) significant differences between AgNPs from the same growth phase but medium with different chlorine concentration; and (•) significant differences between AgNPs from the same growth phase and medium with the same chlorine concentration but different pH. *p*-value < 0.05.

**Table 3:** Antibacterial activity of AgNPs harvested at several times during reaction.

AgNPs	H. T. (h)	*E. coli* ATCC 25922				*S. aureus* CECT 108			
		MIC (µg/mL)	MBC (µg/mL)	IC_50_ (µg/mL)	ICb_50_ (µg/mL)	MIC (µg/mL)	MBC (µg/mL)	IC_50_ (µg/mL)	ICb_50_ (µg/mL)
**L4Cl-AgNPs**	**24**	0.23	0.46	0.13 ± 0.01 *	0.18 ± 0.04	0.93	2.78	0.65 ± 0.02 *	0.89 ± 0.11
	**48**	0.55	1.09	0.20 ± 0.01	0.36 ± 0.01	2.18	>2.18	0.78 ± 0.01	0.92 ± 0.13
	**72**	0.44	0.87	0.26 ± 0.03	0.26 ± 0.02	1.74	>1.74	0.79 ± 0.06	1.13 ± 0.04
	**144**	1.59	>1.59	0.35 ± 0.07	0.70 ± 0.15 *	6.12	>6.12	1.98 ± 0.02 *	2.88 ± 0.54 *
**L4-AgNPs**	**48**	1.49	1.49	0.42 ± 0.10 *	0.44 ± 0.05 *	5.95	>5.95	3.19 ± 0.09 *	3.41 ± 0.40 *
	**100**	1.58	1.58	1.14 ± 0.08	1.52 ± 0.38	12.65	25.30	4.64 ± 0.35 *	4.66 ± 0.65 *
	**200**	1.93	1.93	1.17 ± 0.34	1.74 ± 0.29	15.43	30.86	6.41 ± 0.28	7.44 ± 0.06
	**400**	2.24	2.24	1.44 ± 0.44	2.11 ± 0.65	17.90	35.80	9.52 ± 0.05 *	9.61 ± 1.06 *

H.T.: harvest time. (*) indicates significant differences between the values of IC_50_ or ICb_50_ and the same values of AgNPs harvested at 72 h for L4Cl-AgNPs and 200 h for L4-AgNPs. *p*-value < 0.05.

**Table 4:** Antibacterial activity of AgNPs from successive rounds of synthesis with the same broth.

AgNPs	Batch	*E. coli* ATCC 25922				*S. aureus* CECT 108			
		MIC (µg/mL)	MBC (µg/mL)	IC_50_ (µg/mL)	ICb_50_ (µg/mL)	MIC (µg/mL)	MBC (µg/mL)	IC_50_ (µg/mL)	ICb_50_ (µg/mL)
**L4Cl-AgNPs**	**S1st**	0.47	0.47	0.14 ± 0.05	0.29 ± 0.05	1.87	3.73	0.72 ± 0.05	0.98 ± 0.11
	**S2nd**	0.42	0.42	0.19 ± 0.02	0.30 ± 0.00	1.69	1.69	0.65 ± 0.01	1.18 ± 0.39
	**S3rd**	0.84	1.84	0.31 ± 0.03	0.42 ± 0.00	3.63	3.63	1.15 ± 0.06 *	1.27 ± 0.19
	**S4th**	1.93	7.73	1.18 ± 0.21 *	1.46 ± 0.14 *	15.47	>15.47	6.90 ± 0.89 *	7.29 ± 0.89 *
**L7Cl-AgNPs**	**S1st**	0.84	0.84	0.43 ± 0.16	0.43 ± 0.01	3.43	6.69	1.53 ± 0.10	2.13 ± 0.35
	**S2nd**	0.84	0.84	0.37 ± 0.07	0.36 ± 0.04	1.68	6.73	1.61 ± 0.07	1.56 ± 0.23
	**S3rd**	2.13	2.13	0.80 ± 0.13	NC	8.50	>8.50	4.62 ± 0.29 *	4.61 ± 0.34 *
	**S4th**	4.26	8.16	3.16 ± 0.24 *	4.57 ± 0.08 *	17.24	>17.24	7.51 ± 0.76 *	8.20 ± 1.25 *

AgNPs from first (S1st), second (S2nd), third (S3rd), and fourth (S4th) syntheses; NC: non-calculated; (*) indicates significant differences between the values of IC_50_ or ICb_50_ of S1st and the same values of S2nd, S3rd, and S4th AgNPs. *p*-value < 0.05.

**Table 5:** Toxicity (IC_30_) of the AgNPs against HCT116 cells.

AgNPs	IC_30__AB (μg/mL)	IC_30__CFDA-AM μg/mL)	IC_30__NRU (μg/mL)
**AgNO_3_**	4.01	3.43	4.76
**E4Cl-AgNPs**	15.25 */#/•	9.28 */#/•	23.78 */•
**L4Cl-AgNPs**	1.59 */#	1.82 */#	2.15 */#/•
**E4-AgNPs**	>25.00 #/•	>25.00 #/•	26.18
**L4-AgNPs**	>25.00 #/•	>25.00 #/•	25.67/#
**E7Cl-AgNPs**	1.94 #/•	3.02/•	2.53 */#/•
**L7Cl-AgNPs**	2.38 #	3.44/#	6.85 */#/•
**E7-AgNPs**	15.14 #/•	5.37 */•	21.22 #
**L7-AgNPs**	20.20 #/•	11.35 */#/•	16.02 #

(*) indicates significant differences between AgNPs prepared from the medium with the same chlorine concentration but different growth phase; (#) indicates significant differences between AgNPs from the same growth phase but medium with different chlorine concentration; and (•) indicates differences between AgNPs from the same growth phase and medium with the same chlorine concentration but different pH. *p*-value: <0.05.

**Table 6:** Antibacterial synergy effects of the AgNPs in combination with classic antibiotics in terms of FICI and MF.

AgNPs	Bacteria	Ap		Cp		Ep		Km		Nx		Sm		Tc	
		FICI	MF	FICI	MF	FICI	MF	FICI	MF	FICI	MF	FICI	MF	FICI	MF
**E4Cl-AgNPs**	***E. coli***	2.000	1	2.000	1	1.000	2	0.094	32	1.000	2	0.063	32	0.375	4
	***S. aureus***	0.375	4	1.000	2	2.000	1	0.180	32	2.000	1	0.188	16	2.000	1
**L4Cl-AgNPs**	***E. coli***	1.000	2	2.000	1	1.000	2	0.125	16	1.000	2	0.094	32	0.500	4
	***S. aureus***	0.375	4	1.000	2	2.000	1	0.180	16	1.000	2	0.125	16	2.000	1
**E4-AgNPs**	***E. coli***	2.000	1	2.000	1	1.000	2	0.094	32	2.000	1	0.125	16	0.375	4
	***S. aureus***	0.375	4	0.750	2	2.000	1	0.180	16	2.000	1	0.156	8	1.000	2
**L4-AgNPs**	***E. coli***	1.000	2	1.000	2	0.750	4	0.063	32	0.750	2	0.094	16	0.500	4
	***S. aureus***	0.500	4	0.750	2	2.000	1	0.125	16	0.750	4	0.063	32	0.750	4
**E7Cl-AgNPs**	***E. coli***	2.000	1	1.000	2	2.000	1	0.094	32	2.000	1	0.313	16	0.250	8
	***S. aureus***	0.250	8	0.625	2	2.000	1	0.125	16	1.000	2	0.125	16	1.000	2
**L7Cl-AgNPs**	***E. coli***	1.000	2	2.000	1	1.000	2	0.063	32	0.750	2	0.047	32	0.375	4
	***S. aureus***	0.375	4	1.000	2	2.000	1	0.180	16	0.625	8	0.094	32	1.000	2
**E7-AgNPs**	***E. coli***	0.625	8	1.000	2	0.750	4	0.156	32	1.000	2	0.125	16	0.500	4
	***S. aureus***	0.625	8	2.000	1	2.000	1	0.375	4	2.000	1	0.125	16	2.000	1
**L7-AgNPs**	***E. coli***	1.000	2	2.000	1	2.000	1	0.156	32	1.000	2	0.156	32	0.188	8
	***S. aureus***	0.500	2	1.000	2	2.000	1	0.125	16	2.000	1	0.125	16	1.000	2

Test bacteria: *E. coli* ATTC 25922 and *S. aureus* CECT 794. Ampicillin (Ap); Ciprofloxacin (Cp); Ertapenem (Ep); Kanamycin (Km); Nalidixic Acid (Nx); Streptomycin (Sm); Tetracycline (Tc).

## Data Availability

Data are contained within the article and Supplementary Materials.

## Supplementary Materials

The following supporting information can be downloaded at: https://www.mdpi.com/article/10.3390/ijms242216183/s1.
