# Supplementary material for: Microalga Broths Synthesize Antibacterial and Non-Cytotoxic Silver Nanoparticles Showing Synergy with Antibiotics and Bacterial ROS Induction and Can Be Reused for Successive AgNP Batches
Source: Int J Mol Sci. 2023 Nov 10;24(22):16183. doi: 10.3390/ijms242216183 (PMC10670984; doi:10.3390/ijms242216183)
Supplement: Supplementary file 1 [file ijms-24-16183-s001.zip › ijms-2659206-supplementary.pdf]

Table S1. Susceptibility of *Pseudomonas aeruginosa* strains to AgNPs.

AgNPs	Strain	MIC (μg/mL)	MBC (μg/mL)	IC_{50} (μg/mL)	ICb_{50} (μg/mL)
L4-AgNPs	**CECT 108**	1.84	1.84	0.28 ± 0.04	0.70 ± 0.12
	**PA01**	0.92	1.84	0.14 ± 0.01	0.52 ± 0.10
	**PA14**	0.92	1.84	0.17 ± 0.07	0.49 ± 0.05
L7-AgNPs	**CECT 108**	1.32	2.63	0.40 ± 0.05	0.68 ± 0.19
	**PA01**	1.32	2.63	0.26 ± 0.09	0.46 ± 0.03
	**PA14**	1.32	2.63	0.22 ± 0.02	0.57 ± 0.08

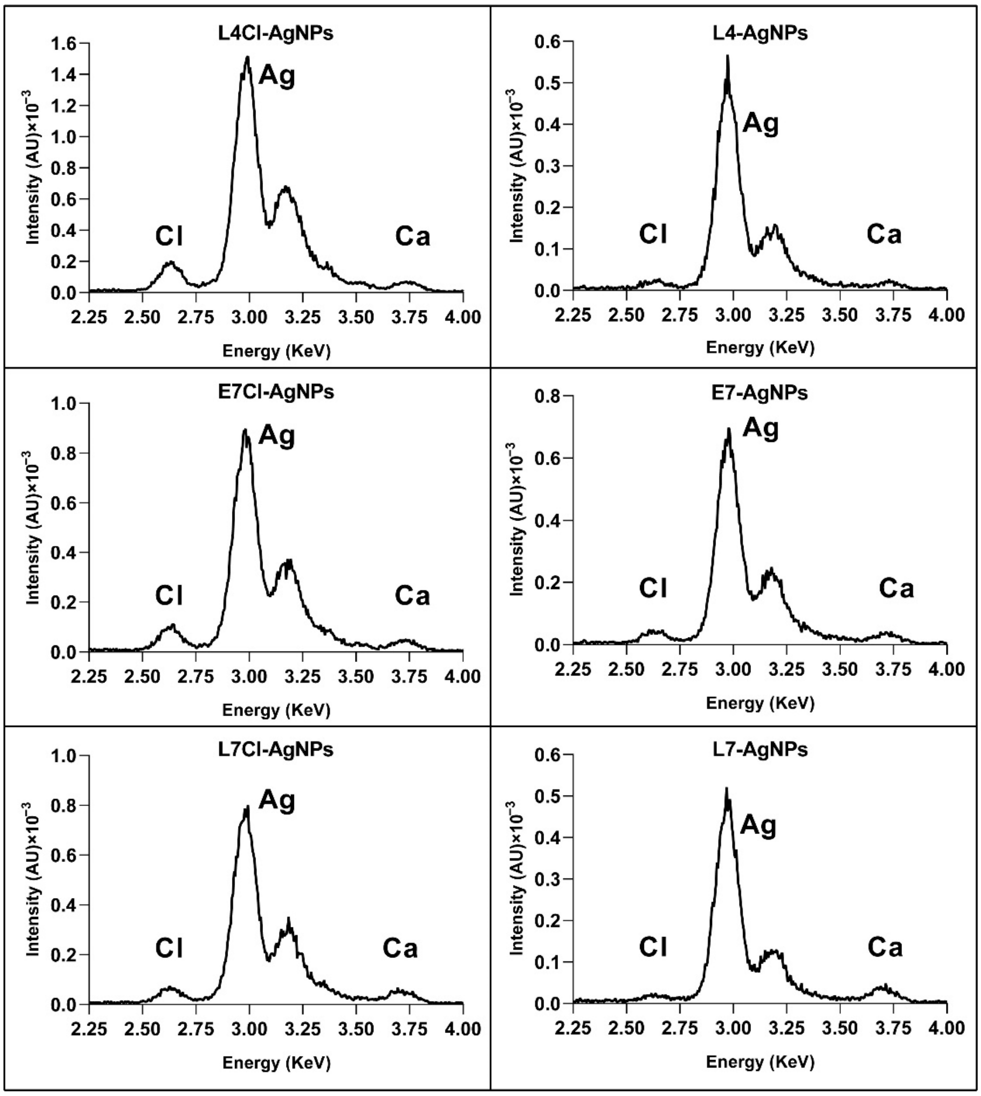

Figure S1: TXRF analysis of the elemental composition of the indicated AgNPs.

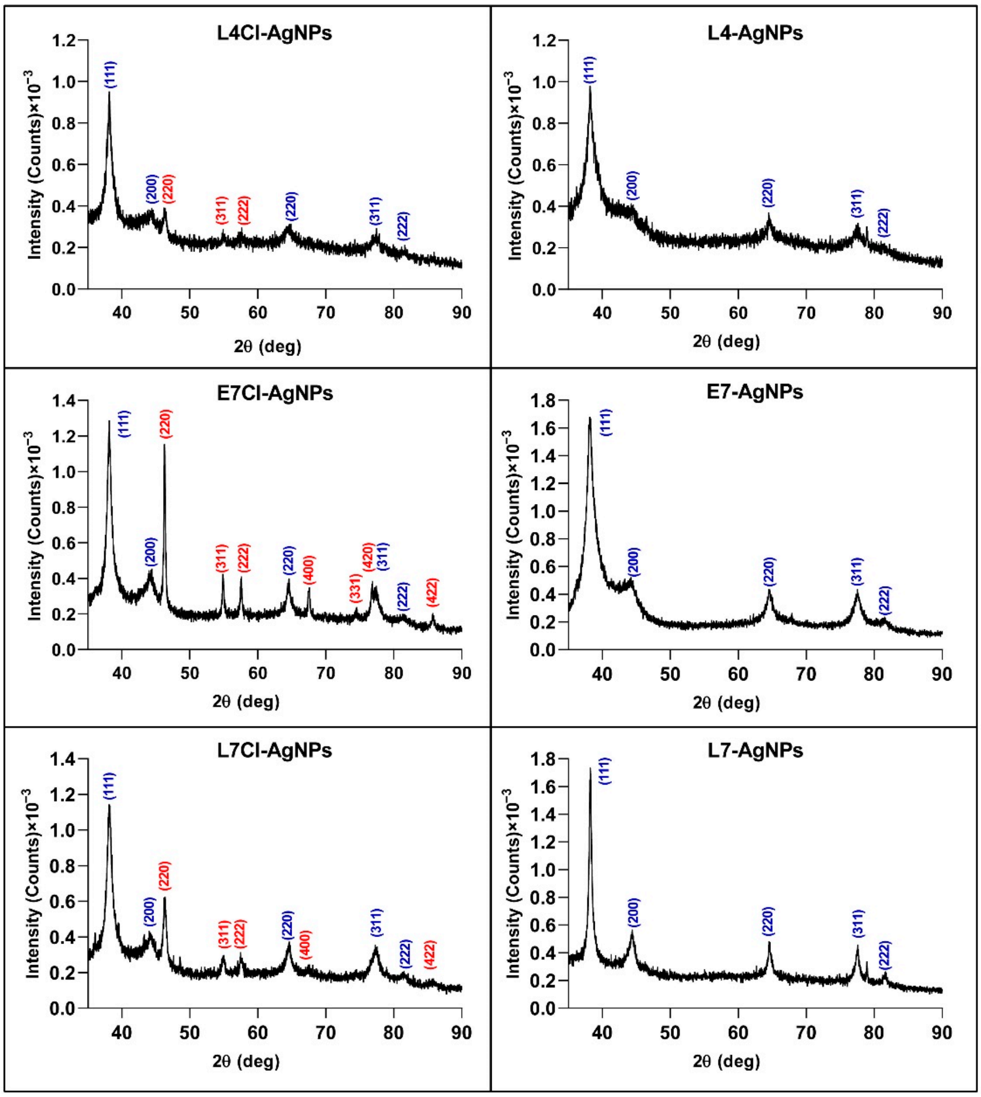

Figure S2: XRD patterns of the indicated AgNPs. Crystals' planes are indicated for Ag^0 in blue and AgCl in red.

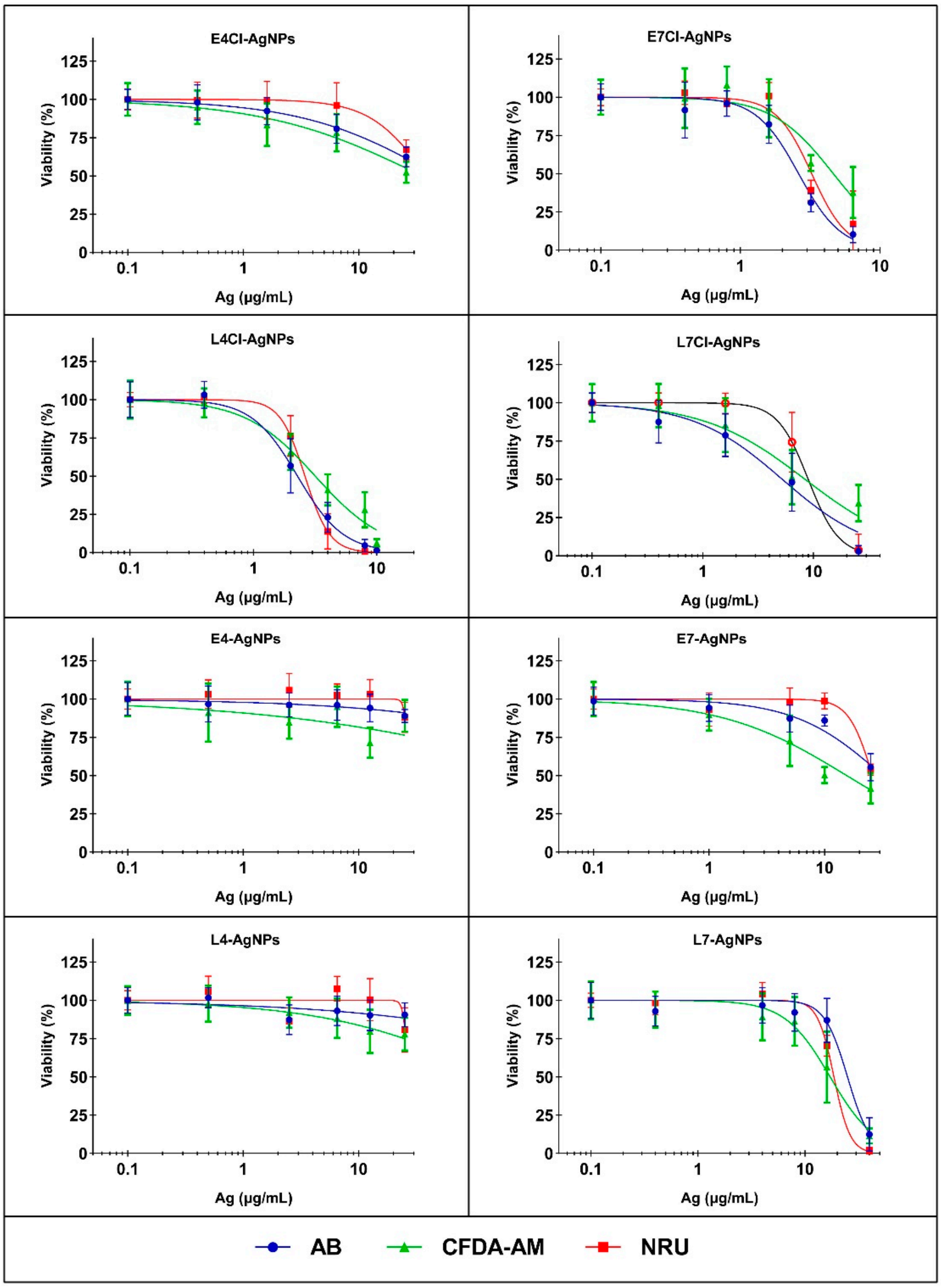

Figure S3: Viability of HTC116 cells in the presence of AgNPs. Blue, the metabolic activity (Alamar blue, AB); Green, the plasma membrane integrity (carboxyfluorescein diacetate acetoxymethyl ester, CFDA-AM); Red, the lysosome integrity (neutral red, NR).

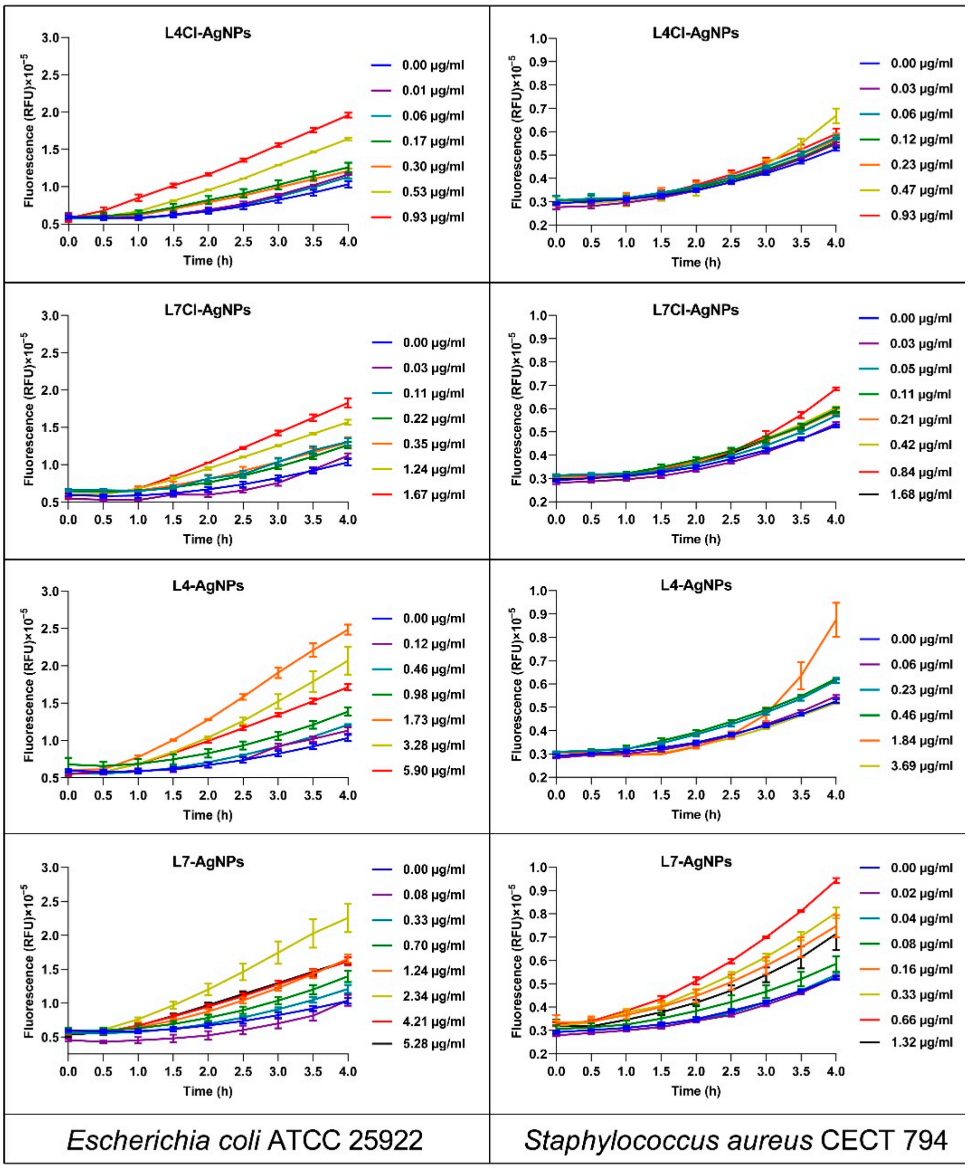

Figure S4: Kinetics of ROS accumulation in the presence of AgNPs.
